# A multiscale analysis of heatwaves and urban heat islands in the western U.S. during the summer of 2021

**DOI:** 10.1038/s41598-023-35621-7

**Published:** 2023-06-13

**Authors:** Kaiyu Chen, Jacob Boomsma, Heather A. Holmes

**Affiliations:** 1 Department of Chemical Engineering, University of Utah, Salt Lake City, UT USA; 2 Department of Atmospheric Sciences, University of Utah, Salt Lake City, UT USA

**Keywords:** Climate and Earth system modelling, Climate-change impacts

## Abstract

Extreme heat events are occurring more frequently and with greater intensity due to climate change. They result in increased heat stress to populations causing human health impacts and heat-related deaths. The urban environment can also exacerbate heat stress because of man-made materials and increased population density. Here we investigate the extreme heatwaves in the western U.S. during the summer of 2021. We show the atmospheric scale interactions and spatiotemporal dynamics that contribute to increased temperatures across the region for both urban and rural environments. In 2021, daytime maximum temperatures during heat events in eight major cities were 10–20 °C higher than the 10-year average maximum temperature. We discuss the temperature impacts associated with processes across scales: climate or long-term change, the El Niño–Southern Oscillation, synoptic high-pressure systems, mesoscale ocean/lake breezes, and urban climate (i.e., urban heat islands). Our findings demonstrate the importance of scale interactions impacting extreme heat and the need for holistic approaches in heat mitigation strategies.

## Introduction

Global climate change is enhancing the intensity and frequency of extreme weather events including heatwaves, which are natural hazards with prolonged periods of excessive heat^1–3^. Excessive heat stress subsequently induces additional risks to both human health and ecosystems^4–8^. At least one-third of the heat-associated mortalities in the past decades have been attributed to climate change^9,10^. It is anticipated that global heat-related mortality will increase from 92,207 to 255,486 from 2030 to 2050 due to climate change^11^. Heatwaves have been attributed to 0.3–0.5% of the European gross domestic product damages and will become five times worse by 2060 if no actions are taken to mitigate climate change impacts^12^.

Many studies have been conducted to scientifically investigate the formation of this natural hazard, especially under the changing climate. Studies in Australia, Europe and the U.S. revealed the dynamic mechanisms and synoptic responses that lead to intense heatwaves. For example, a connection between cyclones/anticyclones and heatwaves was found in Melbourne (Australia), and reinforcing effects were found as a result of the pressure dipole^13^. Heatwaves and their response to vegetation changes were studied in London (U.K.) and central France where heatwave intensity was exacerbated due to vegetation losses^14,15^. Heatwave formation and duration are complex in the western U.S., especially over coastal and mountain areas where heatwave characteristics are strongly affected by marine meteorology and mountainous terrain^16,17^.

In addition to heatwaves, urban heat is one of the most severe heat-related issues urban residents face, often quantified as an urban heat island (UHI). The UHI effect refers to the temperature differences between urban and rural areas caused by a lack of vegetation and increased man-made in highly urbanized areas^18^. While UHI is used to quantify urban heat it does not necessarily quantify the thermal comfort^19^. Additionally, while rural temperatures might be lower than urban they do not always result in decreased heat stress during heatwaves^20^. Urban structure and street canyon geometry in the microscale urban climate zone greatly change the urban surface thermal environment, increasing heat storage^21^. Building materials, such as concrete, absorb heat energy during the daytime and release it back to the surrounding air at night, causing temperatures to stay relatively warmer compared to vegetated areas. Such a phenomenon is frequently observed in many cities^22,23^. Higher urban temperatures lead to negative effects on human health, air quality and ecosystems^24–27^. UHI effects in the U.S. have been widely investigated. Dong et al.^28^ investigated the UHI intensity during an atmospheric blocking event from Aug. 13 to 17, 2007, in Birmingham, AL and found an 8 °C UHI intensity. Across the U.S., UHI intensities in 50 cities were found to be 0.4–2.8 °C^29^. While the UHI provides a method to quantify urban heat, Martilli et al.^19^ point out the importance of mitigating urban heat versus mitigating UHIs. During extreme heat events, mitigating urban heat requires an understanding of both the heatwave and urban climate impacts.

An extraordinary heatwave impacted both the western U.S. and Canada during the summer of 2021 (late June–mid July), with temperature anomalies of ~ 20 °C^30,31^. This event caused significant high-temperature records in many cities^32^. Portland reached 46.7 °C on June 28, breaking its former highest record of 41.7 °C. On the same day, Seattle reached 42.2 °C, breaking its former record of 39.4 °C. Several daily temperature records were also broken in Idaho. The Associated Press reported that more than 116 and 112 heat-related deaths occurred in Oregon and Washington, respectively^33–35^. The majority were in Portland and Seattle where the highest temperatures were well above 40 °C. In Vancouver, British Columbia there were 434 heat-related deaths during this event and one study found increased risks associated with age, sex, and neighborhood characteristics^36^. Extreme heat has been reported in many areas in the western U.S. in recent years, but there have been limited studies that provide a comprehensive analysis of these events, especially across multiple atmospheric scales^37–39^.

In this work, we focus on the Summer 2021 heatwave in the western U.S. to (1) reveal the spatial pattern and magnitude of heatwaves and the connections with the long-term temperature record; (2) systematically investigate the synoptic impacts of heatwave formation; (3) quantitatively assess the impacts and interactions between heatwaves and UHIs in eight major cities. Our overarching aim is to provide a comprehensive analysis across multiple atmospheric scales to better understand extreme heat and its impact on humans and the built environment. Our findings can be used to further assess the potential of heat-related human health risks and to develop policies to mitigate the impacts of increased, acute heat events under a changing climate.

### Long-term temperature variations and extreme temperatures

**Figure 1:**
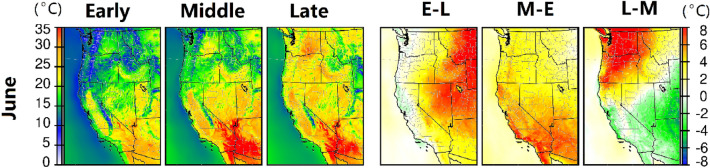
Average 2-m temperature changes in the western U.S. in early summer (June). Early (E), Middle (M) and Late (L) refer to 1st–10th, 11th–20th and 21st to the end of each month, respectively. E–L, M–E and L–M show differences from the previous period. E–L means the differences between the early period of the month and the late period of the previous month. Units are °C*.*

During June 2021, three significant temperature increases were found in the western U.S. (Fig. 1). The increases (> 8 °C, E–L panel in Fig. 1) in early June caused the average temperature in parts of Montana, Wyoming, Utah and Nevada to reach more than 25 °C. Continuous warming in the middle of June led to another significant increase (> 5 °C, M–E panel in Fig. 1) in southern California and western Arizona, with average surface temperatures higher than 35 °C. Temperatures were slightly mitigated in the Southwest during late June (indicated by green in the L–M panel in Fig. 1) when the record-breaking heatwave event occurred in the northwestern U.S. During this heatwave event, the temperature increased by > 8 °C in Washington, Oregon and part of north Idaho and western Montana (L–M panel in Fig. 1). These large temperature changes in June caused extreme heat across the western U.S. In addition to the temperatures, the daytime and nighttime wind and humidity changes are also shown in Figs. S4–S7.

**Figure 2:**
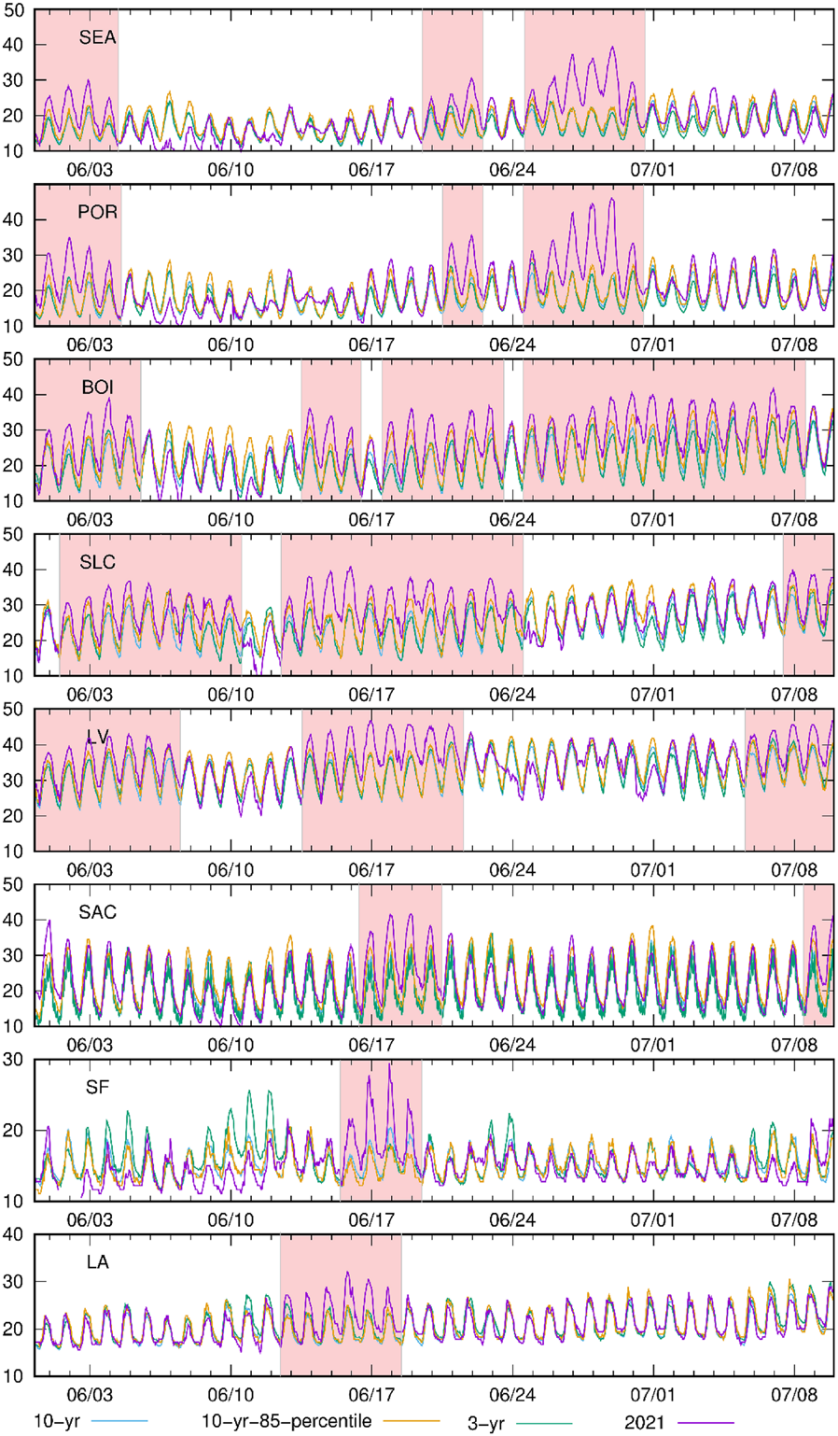
Hourly temperature time series and historical comparisons in 8 major cities in the western U.S. Blue and green lines represent the 10- and 3-year average, respectively. Purple lines show the temperatures in the simulation year (2021). Yellow lines stand for 85 percentile historical (10-year) temperature. City locations are shown in Fig. 6 and units are °C. The light red period indicates a heatwave episode.

While there is no standardized definition for a heatwave, many studies consider a heatwave as a period (> 2 days) with the maximum/mean temperature higher than a reference temperature. The definition of a reference temperature also becomes complicated because heatwaves have intensified and occur more often due to the changing climate^40–44^. Heatwave episodes in this work are defined as continuous days (> 2 days) with the maximum daytime temperature higher than the 85 percentile of the 10-year temperature record (shown in Fig. 2). All of the heatwave events were confirmed by investigating the synoptic conditions, where all had meteorological conditions favorable for heatwave formation. This also corresponds to the spatial temperature patterns shown in Fig. 1. We also use a 10-year and 3-year average to investigate the connections between temperature changes in Summer 2021 and the long-term temperature trend. No significant long-term temperature variation trends are found in cities in the intermountain west, 10-year averages match well with the 3-year average except for slight fluctuations in the middle of June. Boise, Salt Lake City and Las Vegas experienced a hotter early summer, showing less connection with the long-term average temperature, where the daytime temperatures were 5–10 °C higher than the 10-year average.

Significant deviations from the long-term average occurred in southwestern coastal cities (Los Angeles and San Francisco in Fig. 2). There has been a warming trend in the eastern Pacific Ocean in recent years where the sea surface temperatures and Pacific trade winds have also strengthened^45,46^. These effects are shown in Fig. 2, where the average temperatures in the recent 3 years are higher than the 10-year averaged temperatures in Los Angeles and San Francisco, indicating that the increased temperature in these locations is more likely due to the changing climate and the associated El Niño–Southern Oscillation (ENSO) changes in the Pacific Ocean, versus anomalously strong high-pressure systems.

The northwestern U.S. experienced a record-breaking heatwave episode at the end of June. It was formed due to a synoptic-scale high-pressure system, and its formation and setup will be briefly discussed in the next section. The daytime temperatures in Portland were more than 40 °C which is over 15 °C higher than usual. Nighttime temperatures, which are usually around 15 °C, were more than 20 °C. The 2021 temperatures were significantly higher than the long-term trend (e.g., 10-year average) and were primarily caused by an anomalously strong high-pressure system. These anomalous systems and extreme heat can lead to adverse effects on human health^47–49^.

### Heatwave responses to synoptic scale atmospheric dynamics

**Figure 3:**
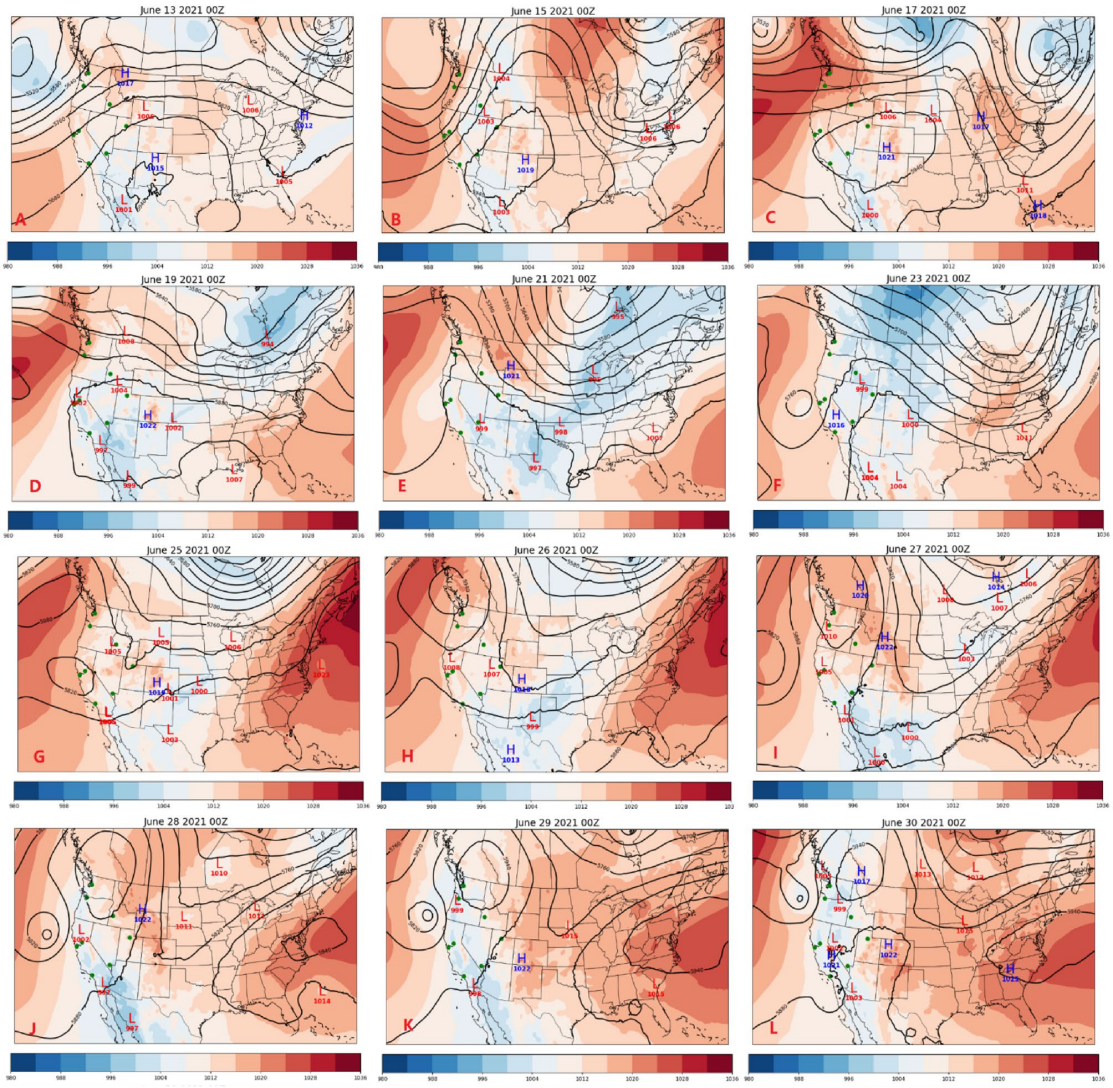
Synoptic meteorology of the heatwave events in June 2021 with 500 hPa geopotential height (black contours) and mean sea-level pressure (MSLP) (shaded color axis). Green markers show the locations of the 8 cities*.*

Heatwaves are typically associated with synoptic-scale atmospheric dynamics^50^. Synoptic scale atmospheric processes are investigated and visualized using daily synoptic weather maps of upper-level (500 hPa geopotential height) and surface (mean sea-level pressure) from reanalysis products. A heatwave occurred over most of the western U.S. in the middle of June, lasting roughly from June 13 (Fig. 3A) to 24 (Fig. 3G). This heatwave began with a large ridge developing over the Intermountain West on June 13. This heatwave led to Salt Lake City reaching the highest temperature (41.7 °C) on June 15 (Fig. 3B). Additionally, widespread daily maximum temperature records were set across the region. By June 17 (Fig. 3C), the ridge flattened, but the air remained warm in the region, with a strong surface high pressure leading to warm air being advected into the Southwest, which sustained the elevated temperatures. The ridge continued to flatten ending the heatwave in several cities by June 19 (Fig. 3D), including most of California. The flattening of the ridge also moved the trough northward, allowing the warmer air mass to reach regions in the Pacific Northwest, resulting in a heatwave in Seattle and Portland. A ridge began to build off the coast of the western U.S. on June 21 (Fig. 3E), while a trough was present over the central U.S. A cutoff low began to form off the west coast, which contributed to the end of the heatwave in California and the Pacific Northwest. Boise and Salt Lake City were unaffected by this development and continued to have elevated temperatures. On June 23 (Fig. 3F), both cities were still in a heatwave, with winds from the south driving the high temperatures in both locations. This trough moved through both cities and ended the heatwave briefly in all locations. At the same time, a large ridge began building off the coast of the western U.S. and continued to build into a strong blocking high pressure. This allowed high pressure to stay nearly stationary and created an intensified Rossby wave pattern, funneling warm air much further north than normal and increasing temperatures. This blocking pattern caused record-breaking temperatures during this heatwave, similar to previous events^51^. This ridge continued to strengthen until the end of June (Fig. 3L) when it began to break, ending the high temperatures along the coast of the Pacific Northwest. The strongest ridge, which occurred on June 28 and 29 (Fig. 3J,K), was correlated with the highest temperatures in Portland and Seattle. The spatial extent of this ridge was relatively small and isolated, which led to other regions in the western U.S. is largely unaffected by this heatwave. Once the blocking pattern broke and began to move downstream, the heatwave began in other regions of the Intermountain West.

**Figure 4:**
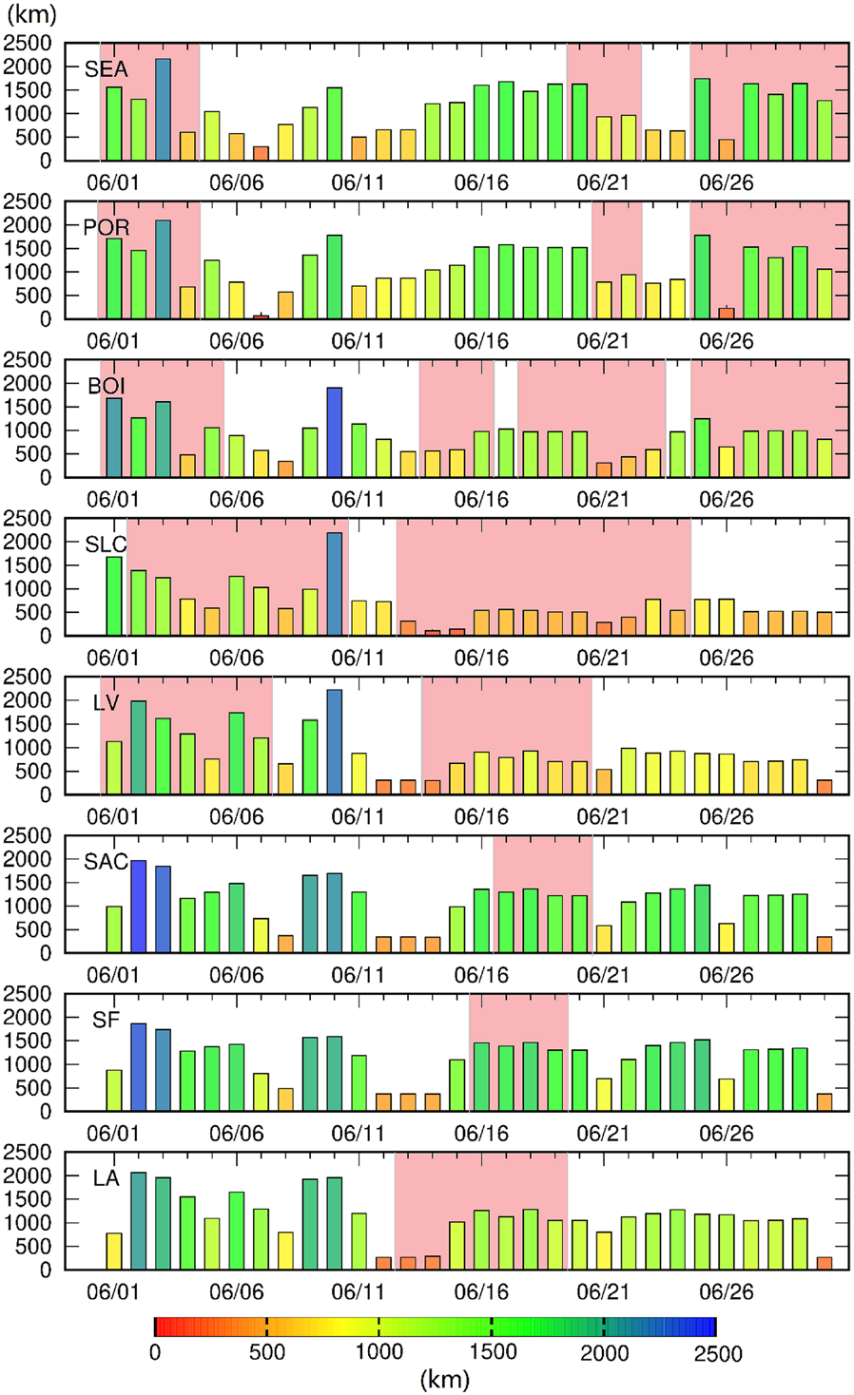
The distances between each city and the center of the nearest surface high-pressure system in June 2021. Units are km. The light red period indicates a heatwave episode. Bar colors represent the distances from close (red) to far (blue).

The center of a surface high-pressure system is typically the region with the most intense subsidence, and, outside of external factors such as advection, would be the region with the highest temperature. Because surface high pressures form downstream of a ridge, it is expected that areas downstream of, or near the center of, a ridge would have the highest surface temperatures. This relationship between the ridge and surface high pressure can be seen in Fig. 3 for the heatwaves. To confirm and clarify these relationships, the distance from the center of the surface high-pressure system (Fig. 4) and the center of the upper-level ridge (Fig. S11) were compared in each of the eight cities. The surface high-pressure systems typically correlate with the highest temperatures, especially for the heatwave episodes that occurred in the middle and end of June. This trend was less apparent in coastal areas, which is likely due to the temperature moderation from the sea breeze. In early June, an upper-level ridge and the resulting surface high-pressure caused heatwaves in Seattle, Portland, Boise, Salt Lake City, and Las Vegas. Once the ridge flattened on June 5, the extended period of high temperatures in Salt Lake City, Boise, and Las Vegas was due to the advection of hot air from the south [e.g., all were upwind of a ridge (Fig. S11) with increased wind speeds (Fig. S12)].

In addition to the synoptic processes, the impacts of recurring climate patterns can also be investigated. Research has shown that the ENSO influences summertime temperatures in western North America both in daily and monthly extremes^52,53^. This indicates that the ENSO pattern could significantly influence the summertime temperatures in these cities, similar to our findings for Los Angeles and San Francisco above. Additionally, there would be a potential increase in occurrences of blocking high pressures in the western U.S. given a negative phase or La Niña conditions^54^. Based on data from NOAA^54^, ENSO was in a negative phase during the Summer of 2021, which might explain these heatwaves climatologically^55^. By contrasting the Summer 2021 temperatures to the 10-year average (Fig. 2), the impacts of the ENSO phase can be investigated. A positive phase of ENSO was present more often over the past 10 years, which is not associated with summer heat extremes over North America^56^. Therefore, during 2021 the negative phase of ENSO and resulting blocking high pressures are clearly shown by the anomalous high temperatures.

### Heatwave impacts, urban climate, and nighttime urban heat island (UHI)

**Figure 5:**
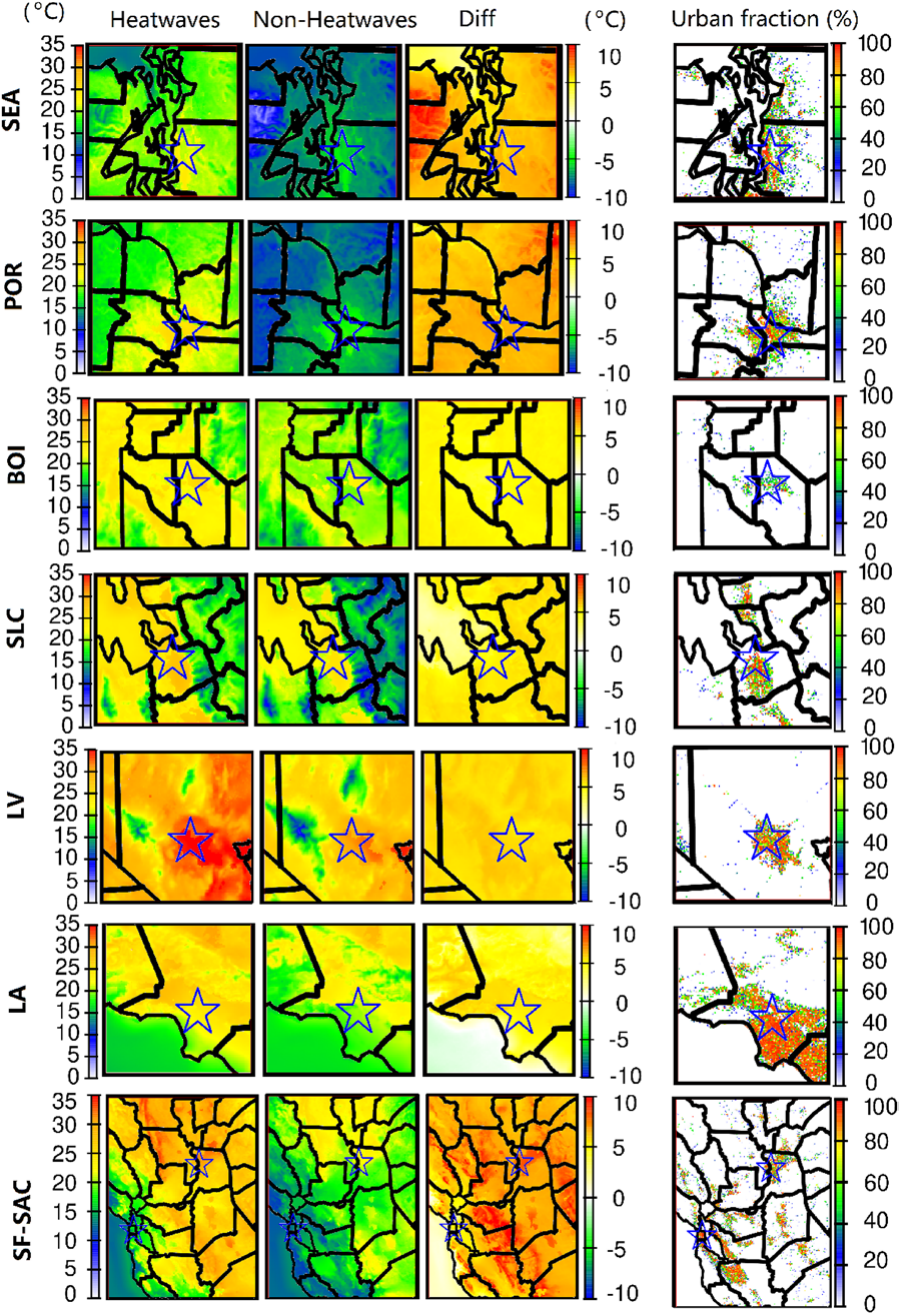
Nighttime temperatures during heatwaves/non-heatwaves periods for June 2021 and urban fractions in eight major cities. Diff refers to the differences between heatwave/non-heatwave episodes. Urban fraction data is obtained from WRF simulation based on 2011 National Land Cover Database. Blue stars indicate the approximate location of cities.

It has been shown that the synergistic interaction between heatwaves and UHIs leads to stronger urban–rural temperature differences during nighttime^19,57,58^, thus we focus on the nighttime UHI in this study. Nighttime temperatures during the heatwave and non-heatwave episodes are shown in Fig. 5. The temperature differences between urban (blue star regions) and rural areas (surrounding areas) are used to indicate the UHIs. Urban fractions are also plotted to visualize the urban distribution. Urban heat islands can be assessed qualitatively in these cities using the spatial distribution of the nighttime temperature. In most cities, Fig. 5 shows a significant temperature difference (~ 5 °C) between urban and rural areas, indicative of an UHI. Figure 5 also shows that there is no significant UHI in the Pacific coast cities (downtown Los Angeles and San Francisco). However, the eastern and southeastern areas of Los Angeles were minimally affected by UHIs with minor temperature increases.

The wind fields (Fig. S10) can be used to investigate the urban climate in the coastal cities of San Francisco and Los Angeles, where ocean breezes cool the urban environment and eliminate the UHI. Cooling effects along the shoreline were due to the relatively cold air over the ocean being transported to inland areas along with an increase in wind speed. The surface high-pressure system during the heatwave episodes caused a large area of relatively low wind speeds (< 2 m/s) across Los Angeles. However, colder air from the Pacific Ocean was transported to the shoreline areas with wind speeds of ~ 3 m/s and ~ 5 m/s for the heatwave and non-heatwave episodes, respectively. The sea breeze has a strong cooling effect, especially during the nighttime for coastal cities, which also mitigates the increased temperature impacts during heatwaves. Previous studies have shown that further away from the ocean, warmer air will remain in urban Los Angeles^59^. This finding was also found in several cities near large bodies of water, such as New York City and Chicago^20,60^.

The UHI intensity in each city was quantitatively estimated based on spatiotemporal temperatures and regression fits based on land use type (Table 1). The regression scatter plots (Fig. S9) also help to comprehensively analyze the urban and rural temperature differences and their changes during the heatwaves. Using this method, there was no UHI detected in Los Angeles where the UHII and R values were negative, confirming what was found above based on the qualitative UHI analysis. At the same time, there are very minor UHI effects in San Francisco and Seattle with intensities less than 1 °C. It can be noted that Seattle also had cooling effects due to breezes from Lake Washington and Elliott Bay. Most inland cities, including Boise, Salt Lake City, Las Vegas and Sacramento had UHI effects during both heatwave and non-heatwave episodes, with an UHII of more than 2 °C during heatwaves. Heatwaves had less effect on intensifying the UHI in these locations, except in Sacramento but had a more profound effect on increasing the baseline temperatures (T_veg) by 3–6 °C. This can also be seen in Fig. S9 where all the temperatures during the heatwave increase by a similar magnitude, regardless of the urban fraction. In Sacramento, heatwaves increased the UHII by ~ 0.7 °C, with more than a 6 °C increase in baseline temperatures. Interestingly, in Portland heatwaves have lower UHII though the baseline vegetation temperature increased by more than 6.5 °C. This is related to different wind speed patterns during the heatwave in Portland compared to the other cities.

**Table 1:** Nighttime urban heat island intensity (UHII) during heatwave episodes in major cities.

City	UHII (°C)		T_veg (°C)		R		Max ∆T (°C)	
	HW	Non-HW	HW	Non-HW	HW	Non-HW	HW	Non-HW
BOI	2.04 (± 0.42)	2.07 (± 0.41)	23.35	20.58	0.55	0.56	6.29 (± 2.89)	6.08 (± 2.41)
LA	− 0.52 (± 0.25)	− 0.17 (± 0.18)	25.54	21.51	− 0.27	− 0.21	9.26 (± 1.92)	6.75 (± 1.75)
LV	2.15 (± 0.23)	1.81 (± 0.22)	32.85	28.78	0.59	0.57	8.48 (± 2.46)	8.94 (± 3.87)
POR	1.51 (± 0.28)	2.52 (± 0.26)	20.06	13.37	0.52	0.66	6.95 (± 2.19)	7.68 (± 3.78)
SEA	0.88 (± 0.12)	0.99 (± 0.09)	19.09	13.59	0.50	0.60	6.04 (± 2.21)	4.08 (± 1.29)
SF	0.74 (± 0.57)	0.80 (± 0.26)	18.84	15.66	0.45	0.66	5.00 (± 2.73)	1.71(± 0.71)
SLC	2.53 (± 0.29)	2.52 (± 0.28)	24.18	21.12	0.58	0.59	11.03 (± 3.23)	12.39 (± 2.17)
SAC	2.06 (± 0.24)	1.38 (± 0.19)	26.19	19.91	0. 61	0.58	8.80 (± 1.72)	6.45 (± 1.31)

UHI strength was estimated from two methods using average nighttime (6 pm to 6 am (+ 1)) temperatures during heatwaves (HW) and non-heatwaves (non-HW) episodes. UHII and T_veg refer to the slope (standard errors) and intercepts for regression in Eq. (1). Max ∆T is the average (standard deviation) maximum nighttime temperature difference between the urban (urban fraction > 90%) and rural (urban fraction = 0) areas.

Daily UHII and averaged urban nighttime wind speeds (Fig. S12) indicate that increasing UHII is typically associated with decreased wind speeds. Low wind speeds extend the residence time of thermal energy emitted from the urban surface because there is less advection^61^. During heatwaves, low wind speeds are expected due to the high-pressure synoptic conditions (i.e., subsidence). However, in Portland, the nighttime wind speed was ~ 3 m/s during non-heatwave episodes but increased to more than 4.5 m/s during heatwaves (Fig. S10). While this windspeed increase during the heatwave is unexpected due to high-pressure subsidence, the center of the synoptic system is not over Portland (Fig. 4, Fig. S11). Therefore, the average windspeeds are slightly higher during heatwave events than during non-heatwave periods. Additionally, surface temperatures increased more in rural regions compared to highly urbanized areas, which also reduces the urban–rural temperature differences.

UHI strength can also be illustrated by the temperature difference between urban and rural areas. The maximum temperature difference between urban (urban fraction > 90%) and rural (urban fraction = 0%) areas across all cities was more than 6 °C and reached more than 10 °C in Salt Lake City. Mean nighttime temperature differences between urban and rural (Fig. S12) are also used to illustrate the strength of UHIs, showing similar variation trends as our UHII results. Quantifying UHI strength is complex and difficult since there are uncertainties in measuring the absolute urban–rural temperature differences. Additionally, the UHI is dynamic and difficult to describe and quantify in a simple index. We analyzed the UHI using multiple methods to describe the UHIs and their characters in each city and to compare the UHI strength across cities. In addition, uncertainties also exist in the dataset used in this study. The urban fraction in this model is from the NLCD 2011 and it is anticipated that urban fractions have changed significantly in the recent decade. The latest version (NLCD 2019) was released but is still unavailable for the atmospheric model used in this study (e.g., WRF). It also should be noted that we investigated the UHI changes during heatwave episodes as defined in Fig. 2, where we assume the temperature changes during this period are mainly due to the heatwaves. The effects of precipitation and cloud cover are minimal in this study (Fig. S13). More development is needed to better describe the urban temperature distribution with a consistent UHI metric.

## Discussion

Several heatwaves impacted the western U.S. during the Summer of 2021 under intensifying heat and drought conditions associated with climate change, which led to hundreds of deaths. We leveraged model simulations and reanalysis data to analyze the scale interactions of the synoptic dynamics and urban microclimate during extreme temperature events. We found that heatwaves have stronger effects in inland cities, causing the average temperature to increase by ~ 4 °C and ~ 10 °C for daytime and nighttime, respectively. Similarly, due to ocean/lake breezes, the UHII indicates that no significant urban heat island effect occurs in Los Angeles, San Francisco and Seattle. For inland cities, the UHIIs are approximately 2 °C (with urban–rural temperature differences of approximately 6–12 °C depending on the city) and the UHII was not significantly changed by heatwaves. This is because heatwaves have stronger temperature effects in rural areas due to the synoptic scale of the high-pressure systems that lead to low wind speeds, decreased humidity and elevated temperatures across a large area (e.g., thousands of km). The uniform temperature increases across both rural and urban areas during heatwaves confirm the importance of mitigating urban heat and not the UHI as suggested by Martilli et al.^19^. To isolate the specific contributions, e.g., urban vegetation and lake/sea breezes, on urban temperatures, future modeling investigations can be designed to focus on model perturbations of these processes so the results can provide a quantitative estimate of their specific impacts on urban heat.

Our findings suggest the importance of considering all atmospheric scales when developing heat mitigation strategies for susceptible communities. The changing climate causes an increase in global temperature and also changes climate patterns (e.g., ENSO) and lake breezes due to drying lake beds (e.g., the Great Salt Lake). Additionally, the built environment and urban microclimates also cause significant temperature increases. Drought conditions have been increasing in recent decades and often lead to vegetation loss in urban areas, which will increase urban temperatures. While high urban temperatures pose a heat-related health risk on a daily basis for urban communities, heatwaves can exacerbate this risk. Heatwaves also lead to significantly elevated health risks for rural communities where the heatwave versus non-heatwave temperature differences in rural regions can be significantly larger than in urban areas. This is because the synoptic scale high-pressure systems cause increased temperatures across large spatial scales and under typical conditions (i.e., non-heatwave) rural areas typically have lower temperatures.

Most heatwaves are associated with strong atmospheric blocking patterns (e.g., high-pressure ridge). However, in the coastal cities, climate patterns that result from sea surface temperatures driven by ENSO also cause increased temperatures, amplifying the presence of widespread heatwaves. Fortunately, in cities near large bodies of water, the UHI and urban temperatures are mitigated by ocean/lake breezes and, in inland cities, it can be mitigated with greening urban infrastructure^62,63^. Heat stress can also be mitigated by increasing green space and the water ecosystem, reducing physical and mental health risks^64^. Considering both microclimate conditions and urban structures in urban development and planning can greatly reduce the potential for heat-related health issues.

## Methods

Extreme heat events and urban climates are important to understand when developing heat mitigation strategies, e.g., urban thermal characteristics and surface morphology^19^. We developed a framework to assess the urban temperature impacts associated with atmospheric processes that occur across multiple scales, in particular, heatwaves, synoptic and mesoscale meteorology, and urban climate. The aim is to understand these processes as a coupled system in an effort to better inform future studies that aim to develop mitigation strategies.

### Meteorological observations

**Figure 6:**
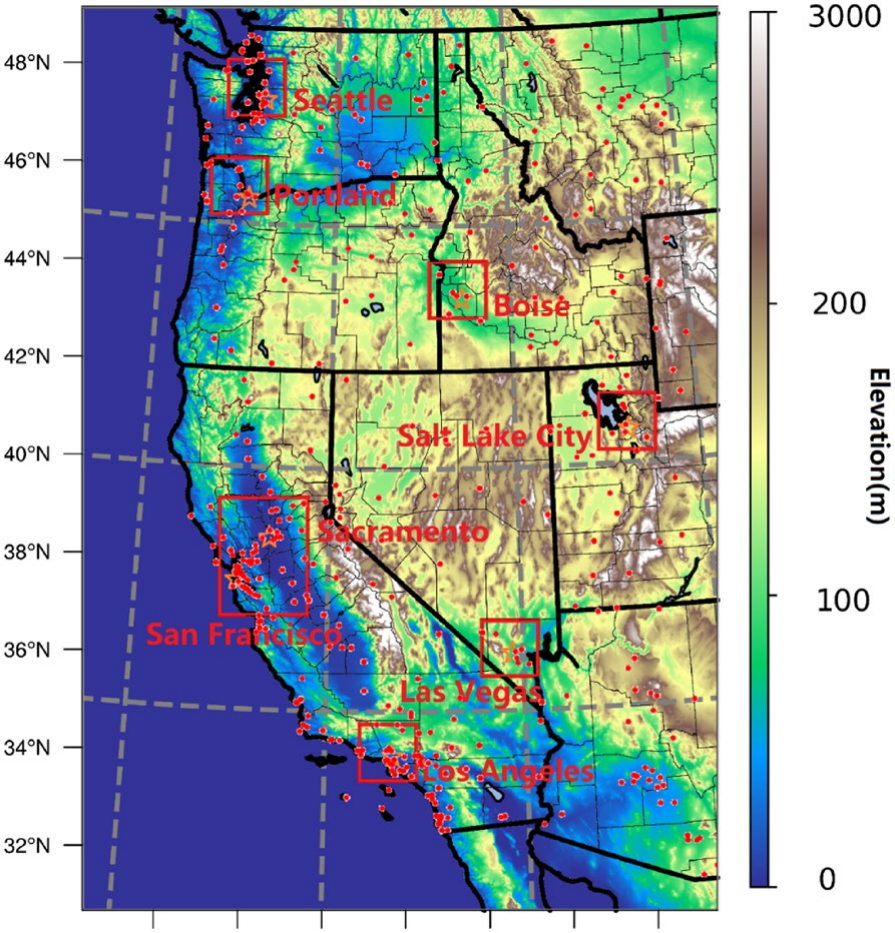
Topographic map, WRF domains, major cities (orange stars), and meteorological observations (red dots). The coarse outer domain (entire figure, 4 km horizontal resolution) with seven nested finer resolution domains (red squares, 1 km horizontal resolution) covers the 8 major cities in our study, including SEA (Seattle), POR (Portland), BOI (Boise), SLC (Salt Lake City), LV (Las Vegas), SAC (Sacramento), SF (San Francisco) and LA (Los Angeles). Map was generated using the NCAR Command Language (NCL).

Observed meteorological data [temperature (T), relative humidity (RH), wind speed (WS) and wind direction (WD)] from the National Climatic Data Center (NCDC)^65^ were used to calculate the long-term temperature trends. This was done by calculating 3-year and 10-year averages and 85th percentiles to compare with the 2021 temperatures in eight major cities. These observations were also used to evaluate the model performance. The observations were averaged across locations if multiple stations were found. There is a total of 396 stations located in the simulation domain, the location of these stations (red dots) can be found in Fig. 6. Additional temperatures observations from MesoWest^66^ were used to evaluate the nighttime urban–rural temperature difference to compare with the model outputs. Four urban sites (with urban fraction > 90%) and rural sites (urban fraction = 0) from four directions to the city center were used to obtain average nighttime temperature differences in 8 major cities.

### Synoptic heatwave analysis

Synoptic scale atmospheric processes drive the conditions that are favorable for heatwave formation. Subsidence that results from an upper-level ridge leads to less vertical mixing, which allows for the surface temperature to rise. Addtionally, surface high-pressure systems form downwind of an upper-level ridge. These surface high-pressure systems also have calm winds, which similarly allows for rising surface temperatures. To characterize the synoptic processes impacting the western U.S., the mean sea level pressure (MSLP) and 500 hPa geopotential height were used. These variables were obtained from the National Centers for Environmental Prediction’s (NCEP) North American Mesoscale Forecast System (NAM) analysis products^67^. The results were used qualitatively with visualizations to confirm the presence of heatwaves during the Summer of 2021. Additionally, to quantify the locations of ridges in the upper-level data and high-pressure systems in the surface data numerical methods were developed using Python. Spatial gradients were calculated using the discretized 500 hPa geopotential height and the MSLP, then spatial maxima were used to determine the locations of ridges and surface high pressures, respectively. These locations represent where, theoretically, there would be the highest surface temperatures based on synoptic conditions alone, and where a heatwave would likely be present.

### Numerical weather prediction model configuration

A numerical weather prediction (NWP) model was used to bridge the observations and reanalysis products described above. NWP provides increased spatial resolution, compared to the reanalysis products and the limited number of monitoring locations, to investigate microscale atmospheric processes. Based on our previous work^20^, we found that the NWP model performance for urban environments and urban heat islands compares well with observations. Therefore, we use the model results to investigate the urban–rural differences in temperature and the meteorological influences of the built environment. The Weather Research and Forecasting (WRF) model (version 3.8) was used to investigate the impacts of heatwaves during the Summer of 2021 in eight major cities. The model initial and boundary conditions came from NAM 12 km analysis data^55^. The National Land Cover Database (NLCD 2011)^68^ was used to provide the national land cover data, providing the location of urban, vegetation and open water areas. The National Urban Database Access and Portal Tools (NUDAPT)^69^ was used to provide urban canopy parameters, capturing the urban morphological features in meteorological simulation to investigate the urban impacts on the meteorological conditions. This study applied a coarse domain covering the western U.S. with a 4 km horizontal resolution. Additional higher resolution (1 km) nested domains, using initial and boundary conditions from the coarse domain, covered eight major cities in the western U.S., shown in Fig. 6. There was a total of 31 layers with 7 under 1000 m, the lowest layer was 40 m above the surface to improve the model performance in the lowest part of the atmospheric boundary layer. The Noah Land-Surface Model (LSM) and Yonsei University planetary (YSU) boundary layer schemes were used to enable the model to provide better 2-m temperatures and more realistic surface winds^70–73^. The Rapid Radiative Transfer Model (RRTM) scheme was applied for the longwave radiation option and the Goddard Shortwave scheme was selected for shortwave radiation. These model configurations have been used to successfully simulate atmospheric conditions in previous work, and have been shown to successfully capture the meteorological conditions in many parts of the U.S.^20,74–77^. The model performance was evaluated using statistical metrics and compared with suggested benchmarks^78^, including mean bias (MB) and gross errors (GE). Results indicate that the simulation provides statistically reliable predictions of temperature in this research and can be used in further analysis. The model evaluation details are provided in the Supplemental Materials. The mapping of model simulation results was done using the NCAR Command Language^79^ and gnuplot^80^.

### Urban heat island (UHI) analysis

There is no standardized method to quantitatively describe the severity of UHIs, and the magnitude and intensity of a UHI depend on the method of UHI calculation^81^. One method, commonly used in observational studies, is to use the urban–rural temperature difference to quantify the UHI. We use the maximum nighttime temperature differences between urban (urban fraction larger than 90%) and rural (urban fraction is 0) areas to illustrate the maximum UHI intensity (i.e., UHI is strongest at night). The spatial average nighttime temperatures are also shown to illustrate the spatial pattern of the temperature differences.

In addition to using the traditional urban–rural temperature difference to determine the severity of UHI, this work applied an analytical method that has been shown to capture the UHI characteristics (a linear regression estimation) to quantify the urban heat island intensity (UHII)^20,81,82^. This UHII estimation fits the temperatures with the urban fractions (shown in Eq. 1) obtained from the WRF simulation results.

1 $${\text{T\_urban}} = {\text{K}} \times {\text{Fraction\_urban}} + {\text{T\_veg}}$$

T_urban refers to the temperatures in urban areas. K is the fitting slope in the regression function, which is the urban heat island intensity (UHII). T_veg is regarded as the baseline temperature, defined as the temperature where the urban fraction is zero. Pearson correlation (R) was conducted to evaluate the relationship between temperatures and urbanization. R values were calculated when the p value was less than 0.01. Urban fractions were obtained from WRF which interpreted the urban fractions of 50%, 90% and 95% based on different urban categories provided by NLCD (2011) and NUDAPT. There are two main benefits of this UHII method over using an urban–rural temperature difference it (1) provides a more statistically optimized interpretation of the role of urban in affecting temperatures and (2) overcomes the limitations of choosing an arbitrary urban/rural grid.

## Supplementary Information

Supplementary Information.

## Data Availability

WPS/WRF model source codes and associated input datasets are available via https://www2.mmm.ucar.edu/wrf/users/download/get_source.html. We thank the NCAR research data archive for providing initial data for WRF simulation and analysis of synoptical dynamics in this work, data is available via https://doi.org/10.5065/G4RC-1N91. Historical data for model validation and analysis is obtained from National Climate Data Center (https://www1.ncdc.noaa.gov/pub/data/noaa/2021/) and MesoWest (https://mesowest.utah.edu/). NCAR Command Language (NCL) was used to visualize and analyze data in this work, which is available via https://www.ncl.ucar.edu/.
