# Supplementary material for: A multiscale analysis of heatwaves and urban heat islands in the western U.S. during the summer of 2021
Source: Sci Rep. 2023 Jun 13;13:9570. doi: 10.1038/s41598-023-35621-7 (PMC10264438; doi:10.1038/s41598-023-35621-7)
Supplement: Supplementary file 1 — Supplementary Information. [file 41598_2023_35621_MOESM1_ESM.docx]

Supplementary Materials

For

A multiscale analysis of heatwaves and urban heat islands in the western U.S. during the summer of 2021

Kaiyu Chen^1^*, Jacob Boomsma^2^ and Heather A. Holmes^1^

^1^Department of Chemical Engineering, University of Utah, Salt Lake City, UT, USA

^2^Department of Atmospheric Sciences, University of Utah, Salt Lake City, UT, USA

*Corresponding author

**WRF Evaluation.** WRF hourly simulations of T, RH, WS and WD are compared with observations obtained from NCDC. Statistical metrics of mean observations (Obs), mean predictions (Pre), mean bias (MB) and gross errors (GE) are calculated for different domain resolutions and months that are shown in Table S1. It is noted that 4km represents the results from the coarse domain (d01) while 1km refers to all smaller inner domains (d02-d08) during the study period. There are a total of 398 monitoring stations are involved in the outer domain providing hourly observations from May to September 2021. Overall, meteorological parameters from WRF simulations match well with observations and all MB are within the. Compared with the coarse domain, the model generally has better performance in higher-resolution domains where the horizontal resolution is 1 km. The mean predicted temperature is only 0.11 ºC lower than the observation which is more accurate compared with MB (-0.22) from the coarse domain. Similar improvements are also observed in other parameters.

Though all MB are within the suggested benchmark criteria, GE of T and WD are significantly higher than the suggested criteria values, indicating the model somehow has less accurate simulations. We further investigate the monthly performance for the high-resolution domain, the results are shown in Figure S1. From the numbers (MB) shown in each panel, it is concluded that the model provides reliable temperatures except in June when the MB significantly exceeds the suggested benchmark of ±0.5 ºC. At the same time, wind direction also has a higher bias in June even though it is still within the suggested benchmark. On the other hand, biases of RH are mainly from September where the MB is -4.25 compared to others that are all within ±2%. Comparisons between urban and rural simulations reveal a better performance in urban areas (Figure S2). MB in urban areas is mostly lower than the suggested benchmark of ±0.5 ºC except for a slight exceedance in September when MB is -0.69. However, there are two underestimates observed in rural areas, one of them has a significant exceedance of MB (-1.13) in June.

**Table S1. Evaluation of WRF model performance**. Evaluation includes simulations of 2-m temperature (T), relative humidity (RH), 10-m wind speed (WS) and wind direction (WD) in the coarse domain (4km) and high-resolution domains (1km). Units are ºC, %, m/s and degrees, respectively.

| Variables | Obs | Pre | MB | GE | Benchmark* |
| --- | --- | --- | --- | --- | --- |
| T_4km | 20.64 | 20.40 | -0.24 | **3.19**** | MB ≤ ±0.5ºC  GE≤2ºC |
| T_1km | 21.30 | 21.18 | -0.11 | **3.15** |  |
| RH_4km | 52.64 | 50.53 | -2.12 | 13.04 | MB ≤ ±10%  GE≤20% |
| RH_1km | 51.51 | 49.75 | -1.76 | 13.25 |  |
| WS_4km | 3.68 | 4.10 | 0.42 | 1.65 | MB ≤ ±0.5 m/s  GE≤ 2 m/s |
| WS_1km | 3.64 | 3.95 | 0.31 | 1.60 |  |
| WD_4km | 218.45 | 216.08 | -2.37 | **56.58** | MB ≤ ±10 º  GE≤30 º |
| WD_1km | 213.33 | 212.52 | -0.81 | **58** |  |

* Obs: mean observations; Pre: mean predictions; MB: mean bias. Benchmarks are obtained from previous studies.

** Bold represents the statistic metrics that exceed the benchmark.


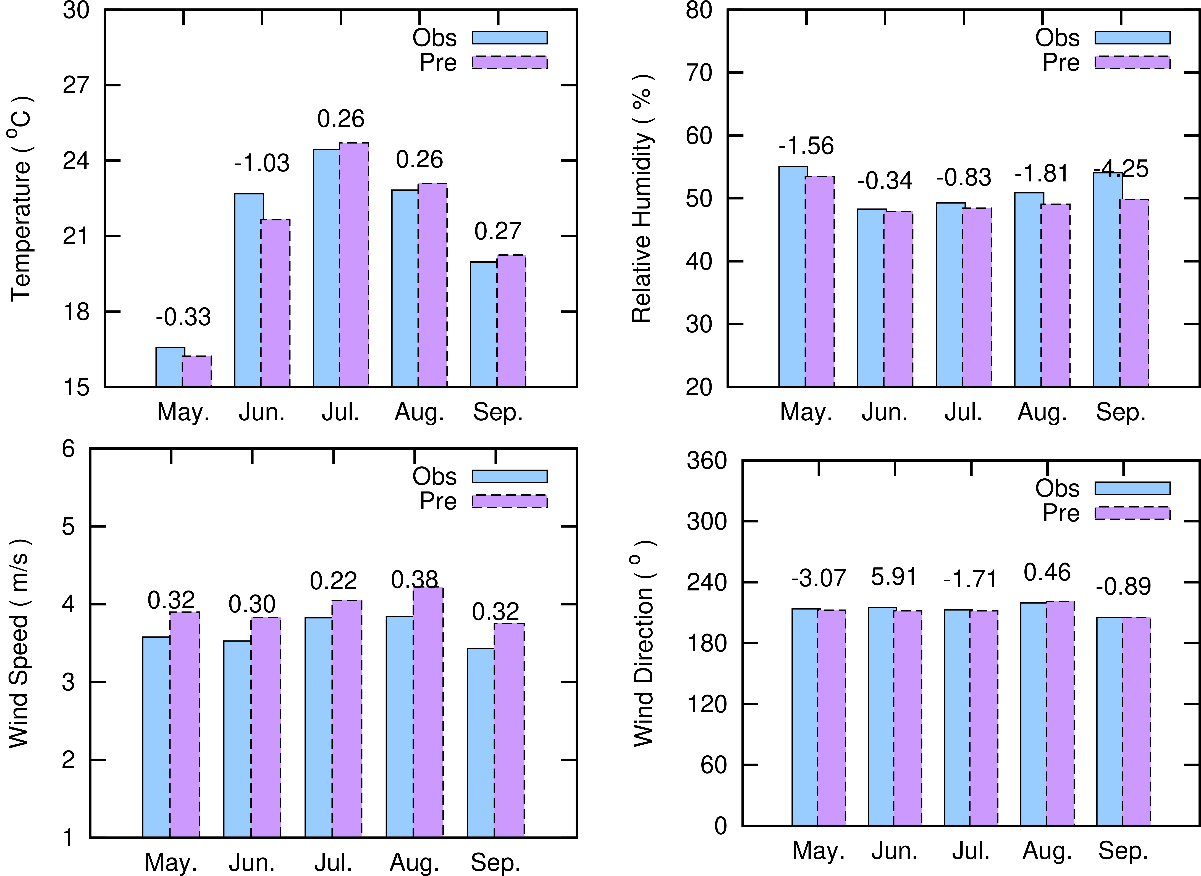


**Figure S1. Monthly model performance for 1km resolution domain.** This evaluation includes temperature, relative humidity, wind speed and direction. Mean biases are shown on the top of each histogram, benchmarks are ±0.5ºC, ±10%, ±0.5m/s and ±10 degree, respectively.


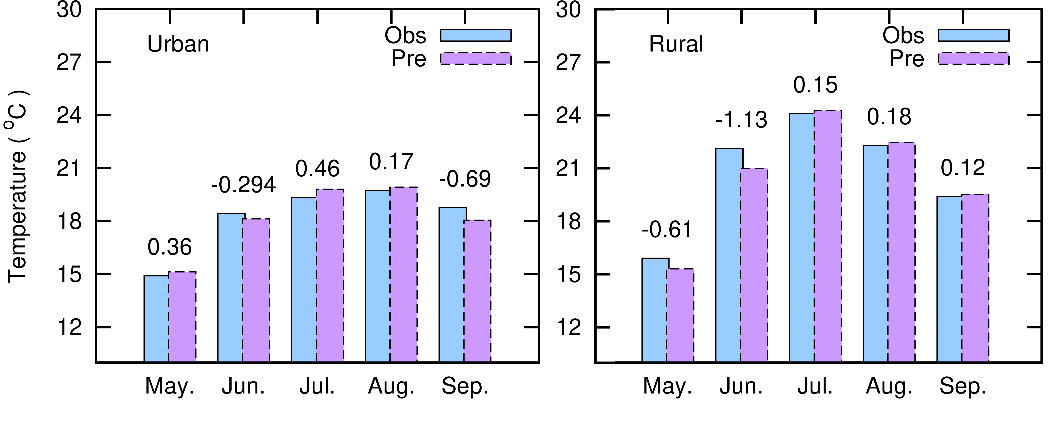


**Figure S2. Monthly model performance of temperatures stratified by urban and rural areas.** Numbers above each histogram bar are the mean bias, the suggested benchmark is ±0.5 ºC.

Hourly temperatures, wind speed and direction in June (when heatwaves occurred frequently in this work) and the comparison with observations are illustrated in the following figures in each city. Most of the monitoring stations from NCDC are located within or around airports, we define the grid with a high urban fraction (>75%) as urban (blue in the figures) and the rural as the grid with a less urban fraction (<30%), other than that, the observation sites were separated out as a third category (red in the figures).

**Seattle** Model provides reliable results in most sites in the Seattle area and slightly overestimates the daytime temperatures on June 12 and June 14 in urbanized areas. Otherwise, the model successfully catches the daily temperature variations and the heatwave impacts during the end of June. In addition, the model has better performance in urbanized areas (urban and airport), agreeing better with the observation while the simulations in rural areas tend to overestimate the daytime maximum temperatures by 3-5 ºC. The overall temperature MB in urbanized and rural areas are -0.28 and 1.25, respectively. In the urbanized area, wind speeds agree well with the observation, with the overall MB less than 0.5 m/s in Seattle. However, the bias is slightly higher in the rural area where the wind bars in 994350-99999 don’t always match well with each other. This result indicates the model statistically provides reliable temperatures in urban Seattle and can be used for further analysis.


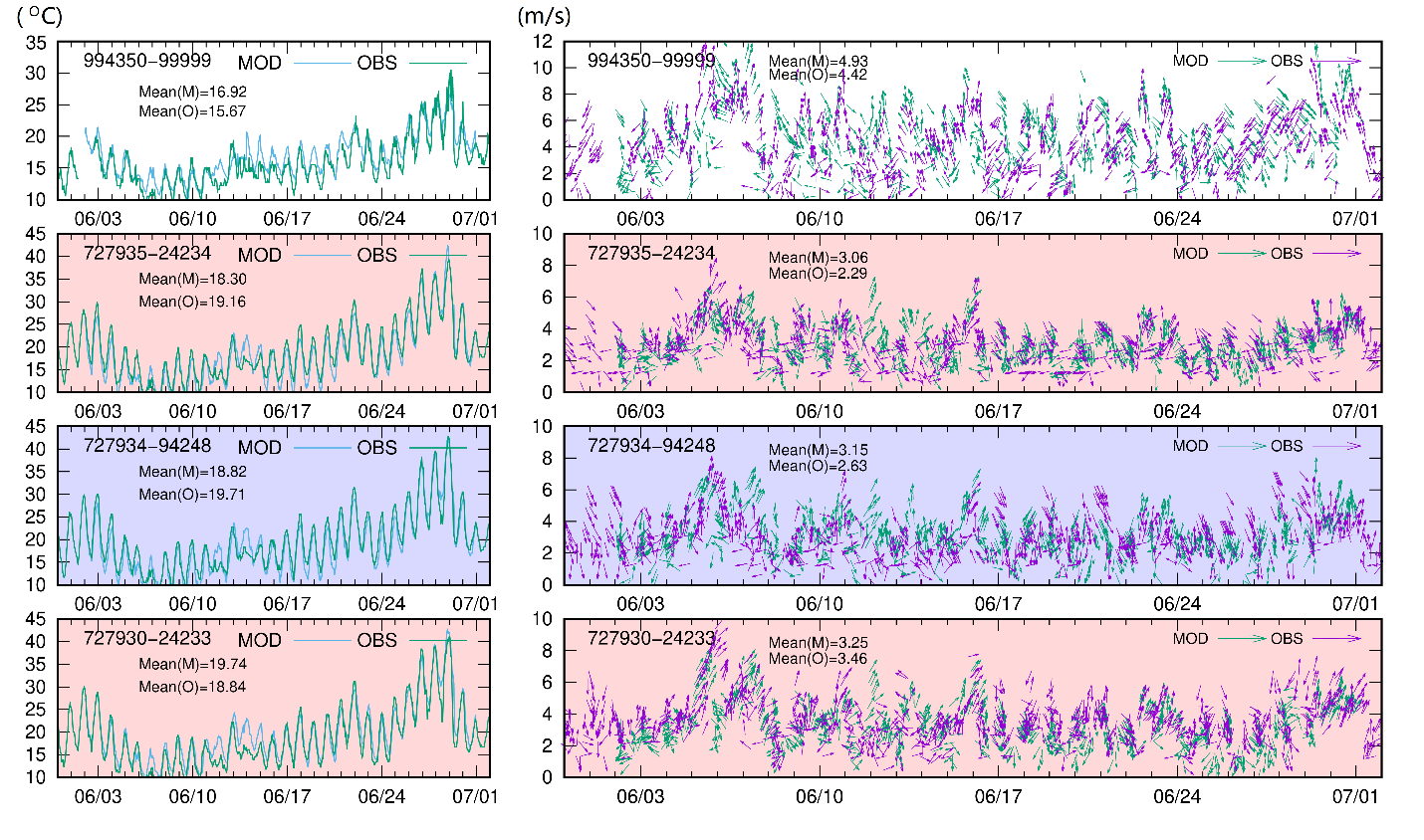


**Figure S3(A) Model performance of temperature (left) and wind (right) time series (June) in rural and urbanized areas in Seattle.** White indicates rural stations, blue is urban and red is sites located near airports. Station IDs are shown in each panel with the mean value of model simulations (M) and observations (O). Arrows in the right penal indicate the wind direction. Units are ºC and m/s, respectively.

**Portland** Model sightly underestimates the temperatures at most sites but still captures the diurnal variation and the impacts from heatwaves at the end of June when the daytime temperatures were higher than 40 ºC. Compared to the performance in the rural sites (MB around 2 ºC), the model provides simulation results with lower biases in urban areas, with an overall MB of ~1.6 ºC. The model tends to overestimate the wind speeds in rural areas, the MB is around 1 m/s while it is slightly lower in the airport (overall MB is 0.55 m/s). We investigated the location of these two airports and found the vegetation coverage is relatively high (60%-70%) even though they are close to urban areas. The model has less accuracy in the area with low urban fractions. Further evaluation might be necessary to provide more detailed model performance in central urban areas with other observation datasets.


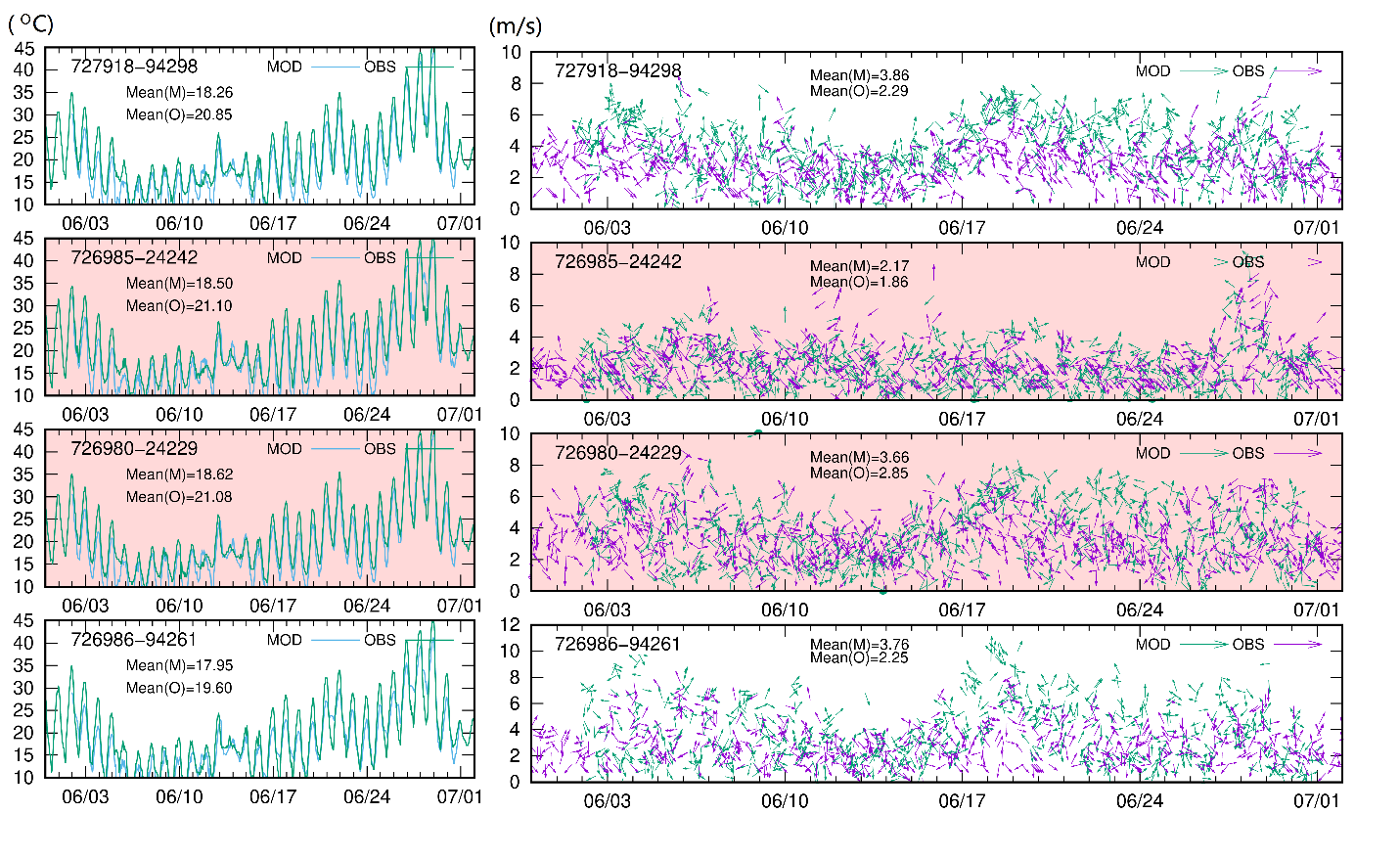


**Figure S3(B) Model performance of temperature (left) and wind (right) time series (June) in rural and urbanized areas in Portland.** White indicates rural stations, blue is urban and red is sites located near airports. Station IDs are shown in each panel with the mean value of model simulations (M) and observations (O). Arrows in the right penal indicate the wind direction. Units are ºC and m/s, respectively.

**Las Vegas** Model simulation of temperatures in urban Las Vegas agrees well with observations, matching the daytime maximum temperatures while slightly underestimating the nighttime minimum temperatures in June. The model has significant biases in rural areas, underestimating the daytime maximum temperatures by 3-5 ºC for site 746140-23112 while slightly overestimating the temperature by 2-5 at two other rural sites. Overall, the simulation captures the temperature variation and the impacts of heatwaves in urban areas. Simulations of wind in urban and airport areas match with the observations, with the MB of 0.75 and 0.59 m/s, respectively. Model performance in the urban area is slightly better than in rural areas, the model successfully captures the urban signal in simulating urban weather conditions.


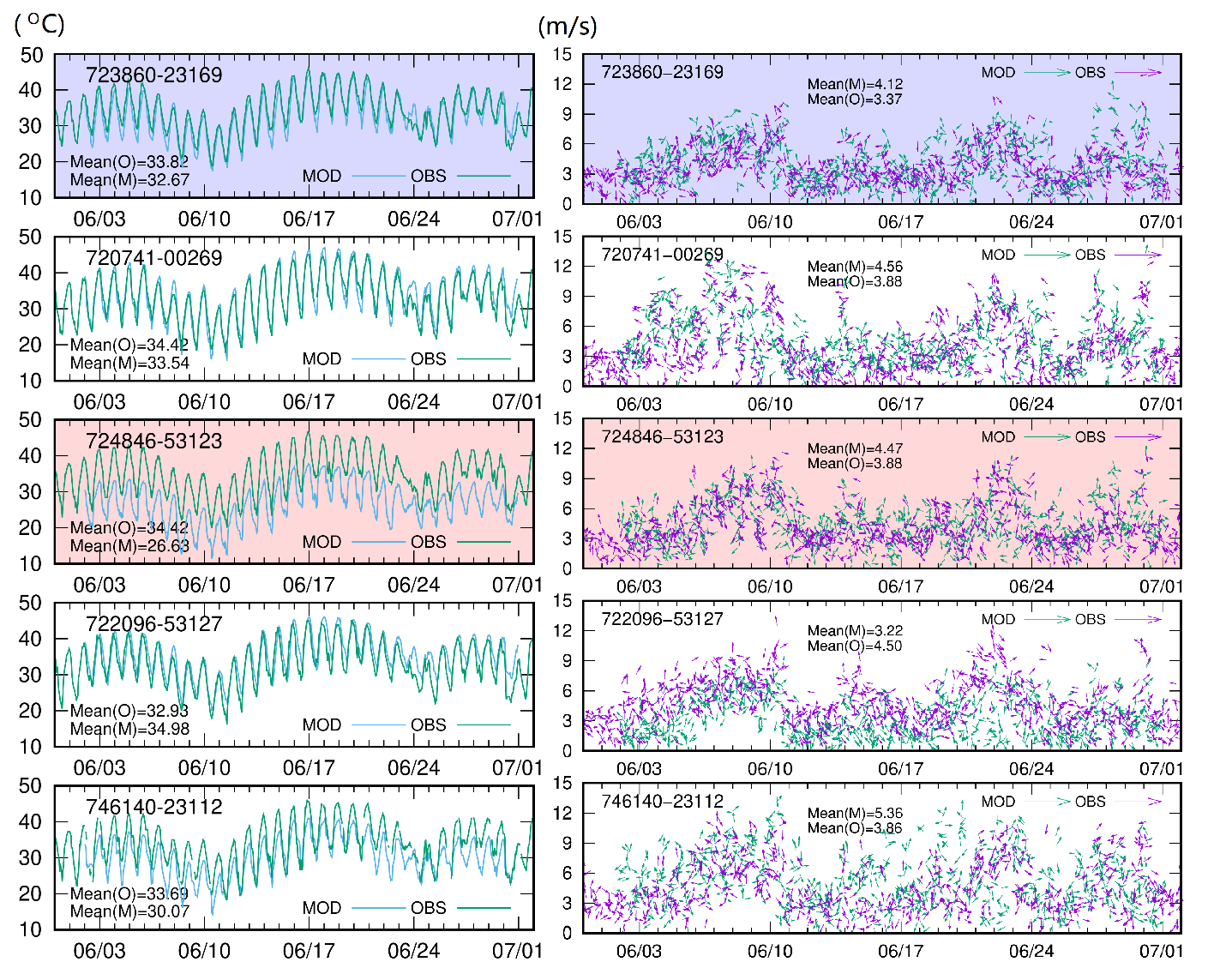


**Figure S3(C) Model performance of temperature (left) and wind (right) time series (June) in rural and urbanized areas in Las Vegas.** White indicates rural stations, blue is urban and red is sites located near airports. Station IDs are shown in each panel with the mean value of model simulations (M) and observations (O). Arrows in the right penal indicate the wind direction. Units are ºC and m/s, respectively.

**Boise** Boise has less population density and most observation sites in this city are away from the downtown area, thus, no urban site can be used to evaluate the model performance in urban areas. However, model performance at the site near the urbanized airport can be used to identify the model capacity in simulating temperatures over urbanized areas. The model provides reliable simulations of temperatures and winds in Boise, the MB at the urbanized airport (720734-00264) station is 0.14 ºC. It is noted that the model underestimates the daytime temperatures at site 726810-24131 which is an airport with less urban fraction. The overall MB at locations with a lower urban fraction is below the benchmark (0.5m/s) while the model tends to overestimate the wind speeds at the urbanized airport location by ~1m/s. In general, model provides reasonable estimations of temperature and winds in Boise.


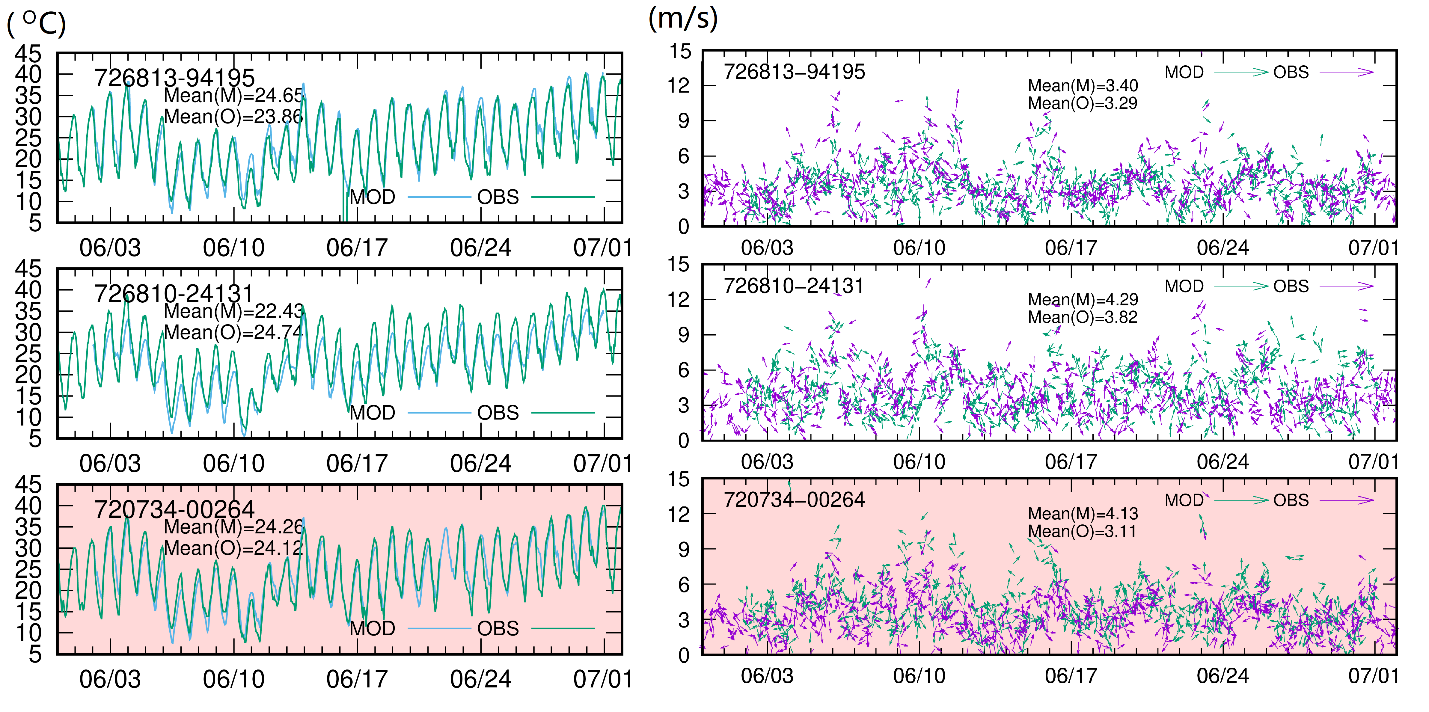


**Figure S3(D) Model performance of temperature (left) and wind (right) time series (June) in rural and urbanized areas in Boise.** White indicates rural stations, blue is urban and red is sites located near airports. Station IDs are shown in each panel with the mean value of model simulations (M) and observations (O). Arrows in the right penal indicate the wind direction. Units are ºC and m/s, respectively.

**Salt Lake City** Observation sites in Salt Lake City are always located in areas with wide vegetation coverage even near the airport. For example, 725720-24127 is located near the SLC international airport which is at the edge of the urban boundary, the grid covers around 50% of unurbanized areas, thus, we identify this site as an airport instead of an urban site. Model performance in Salt Lake City is less reliable in rural areas compared to the other cities in our study. The model fails to simulate the temperature variations at site 720572-99999 which is a rural area near the Great Salt Lake. The problems might be due to the land cover dataset we used in this simulation. We used NLCD 2011 in this work and the lake has been shrinking in recent decades, as a result, there is more lake coverage in this grid in the simulations (compared to real life) so the temperature tends to be flat while the observation shows more fluctuation similar to other inland areas. The model captures the impacts of urbanization at two airport sites where the simulation successfully matches the nighttime temperatures while slightly underestimating daytime temperatures (by ~3 ºC). The model performance of wind simulation matches well with observations, with the MB less than the benchmark of 0.5 m/s, except at site 720565-99999 where the model overestimates the wind speed and thus induces lower estimation of temperatures in this area.


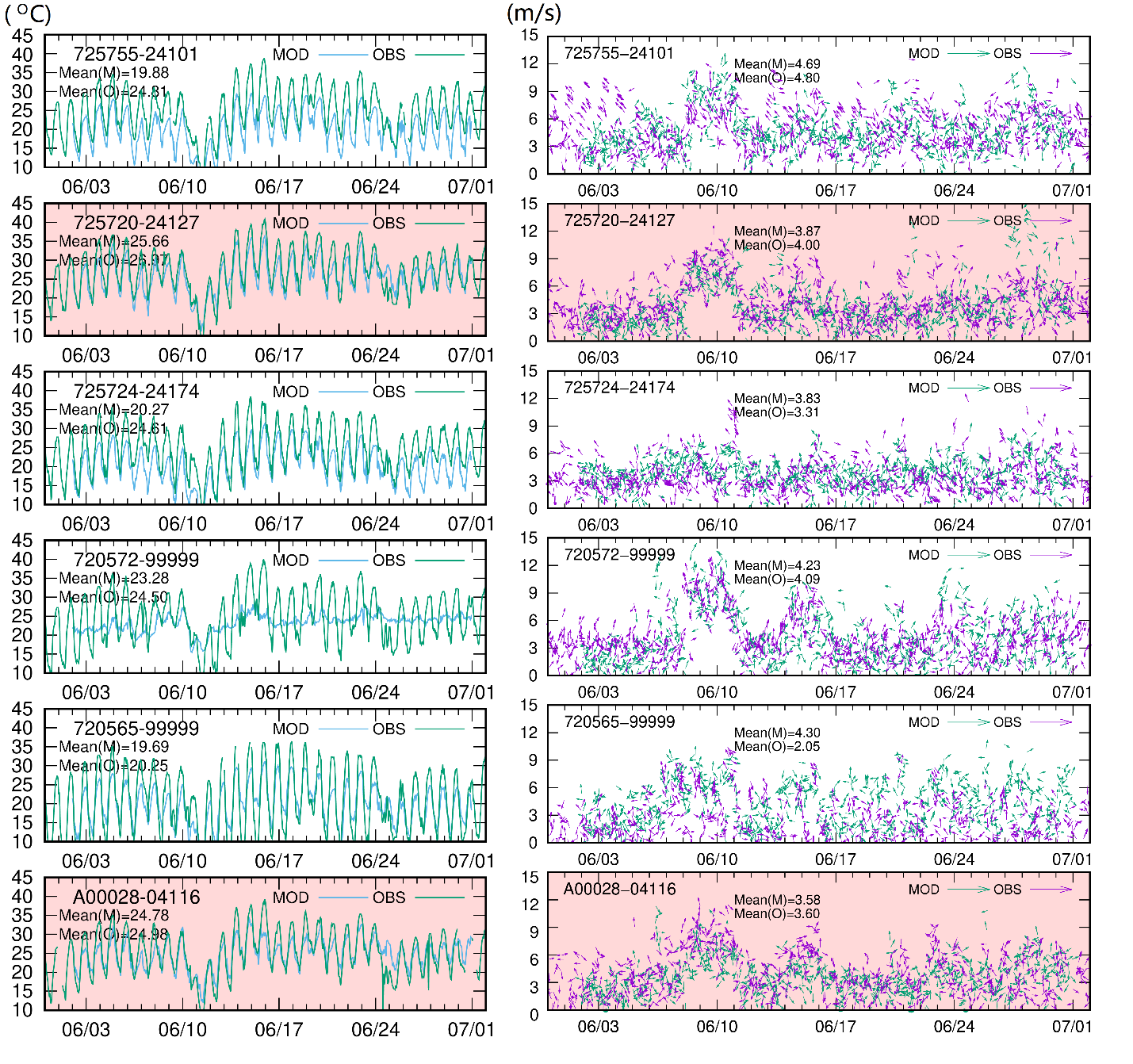


**Figure S3(E) Model performance of temperature (left) and wind (right) time series (June) in rural and urbanized areas in Salt Lake City.** White indicates rural stations, blue is urban and red is sites located near airports. Station IDs are shown in each panel with the mean value of model simulations (M) and observations (O). Arrows in the right penal indicate the wind direction. Units are ºC and m/s, respectively.

**San Francisco** Simulations of temperature and wind in San Francisco do not match well with observations. It is noted that the monitoring stations in San Francisco are always located over water surfaces (e.g., the site near the SFO international airport (724940-23234) and the OAK international airport (724930-23230)) or in higher urbanized areas. In general, temperature simulations in urban areas are usually better than the simulations in less urbanized areas, with the MB ranging from 0.7 to 1.7 ºC. We investigate the reason behind this uncertainty and find the location of monitoring stations in the less urbanized areas to be located on the edge of water/land boundary areas. These locations are where the monitoring data is strongly affected by the sea/land meteorological conditions (e.g. sea breeze) but the model grid, mostly represents the temperature variation of either water or land and fails to match the observations. For example, site 994016-99999 is located over the water surface at Golden Gate Beach while the corresponding grid covers more inland areas, thus the temperatures from the model have more fluctuations while the observations are flat (representing the expected air temperatures over water). The opposite situation is found at the site near the SFO airport (724940-23234), where the monitoring station is located on the edge of the shoreline, and the corresponding grid covers more water area than land area, as a result, the simulated temperature is flat and the observed temperature fluctuates.


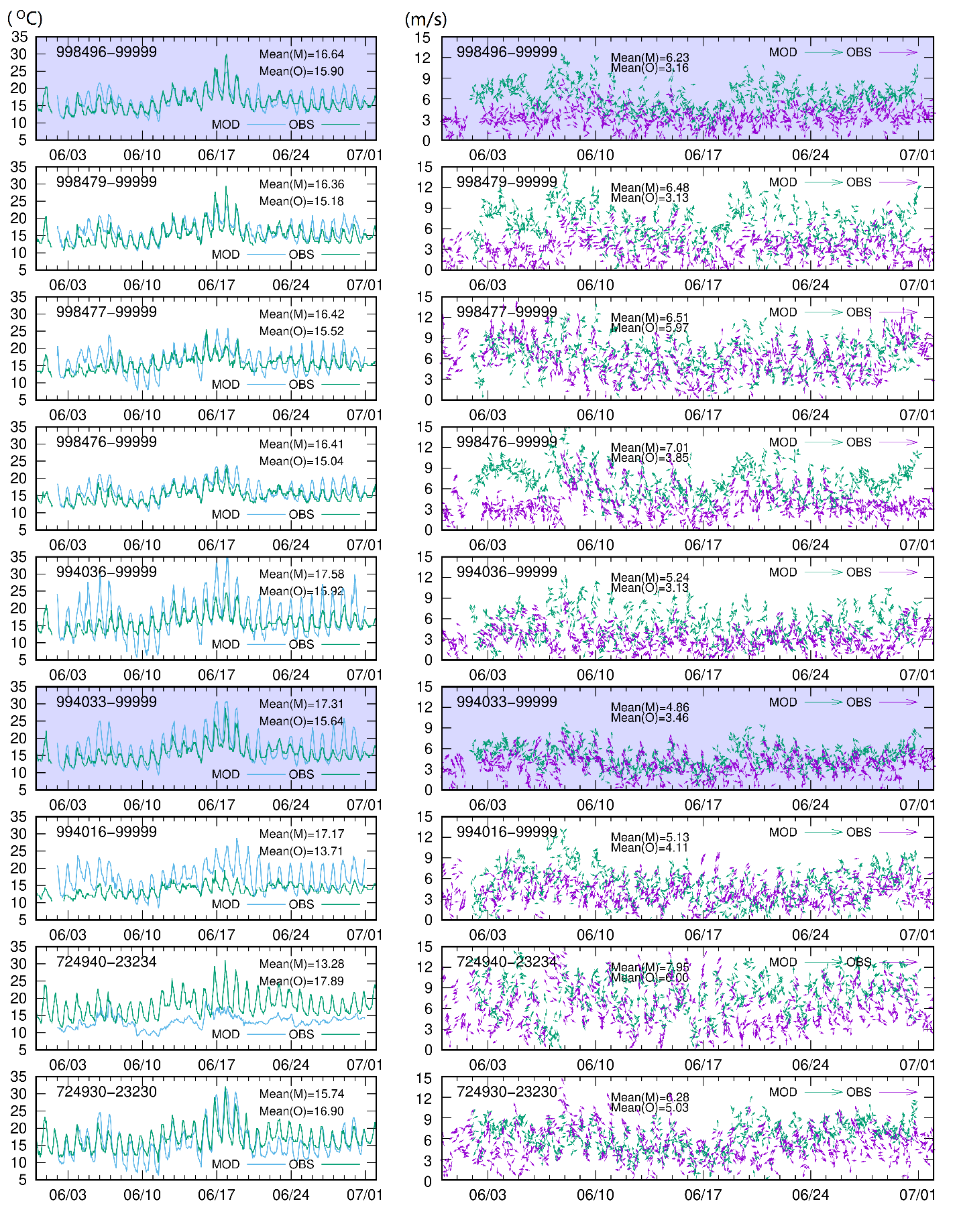


**Figure S3(F) Model performance of temperature (left) and wind (right) time series (June) in rural and urbanized areas in San Francisco.** White indicates rural stations, blue is urban and red is sites located near airports. Station IDs are shown in each panel with the mean value of model simulations (M) and observations (O). Arrows in the right penal indicate the wind direction. Units are ºC and m/s, respectively.

**Sacramento** The model tends to overestimate the maximum daytime temperatures in Sacramento areas by 3-5 ºC and has an overall reliable estimate of nighttime temperatures. The grids with airport monitoring sites near Sacramento have medium urban fractions (40-60%) and are located on the edge of the Sacramento urban boundary (e.g., 724839-93225 at SMF international airport with 60% urban fraction). Thus, these airport sites are classified as urbanized airports instead of urban. the Model performances in urbanized airports and rural areas are similar, the MBs are ~1.6 ºC for each site. Model simulation of wind matches well with observations, with the MB less than the benchmark of 0.5m/s in urban areas.


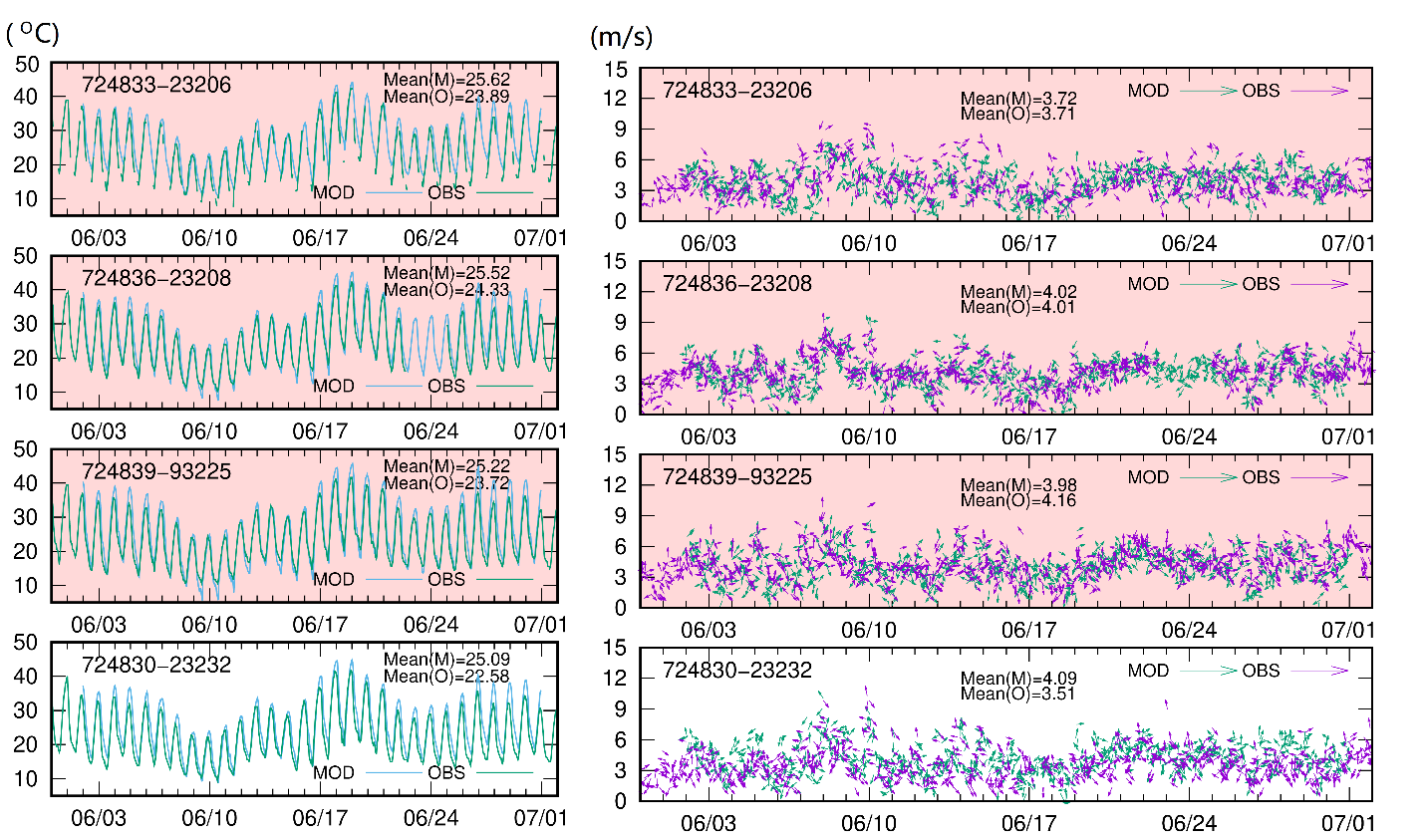


**Figure S3(G) Model performance of temperature (left) and wind (right) time series (June) in rural and urbanized areas in Sacramento.** White indicates rural stations, blue is urban and red is sites located near airports. Station IDs are shown in each panel with the mean value of model simulations (M) and observations (O). Arrows in the right penal indicate the wind direction. Units are ºC and m/s, respectively.

**Los Angeles** Temperature simulations from the model capture the diurnal variations in most sites but overestimate the daytime maximum temperatures by more than 5 ºC. Model simulated temperatures at sites 994028-99999, 722950-23174, and 722885-93197 are much higher than the observations, they show more fluctuations while the observed data tends to have less variability. The biases are due to the model grid configurations. For example, monitoring site 994028-99999 is located on the ocean surface in Santa Monica State Beach, while the corresponding grid includes a more inland area that has a high urban fraction. Temperatures in this grid represent more urban temperatures instead of the temperature changes over the water body, thus the model fails to match the observations at this site. The same situation is found at other sites where the monitoring stations are surrounded by highly urbanized areas (>90%), the model overestimates the urban signal in this case, thus providing higher temperature estimates. To confirm the model performance is reliable, we evaluated the simulated winds at these sites. Model simulation of wind matches well with the observations, with most MBs less than the benchmark of 0.5 m/s except at the sites 722955-03174 and 722950-23174. This result indicates that the model simulations successfully represent the weather conditions in Los Angeles, though there are biases due to the model grid resolution, the result of this simulation can provide reliable temperature diurnal variation, nighttime temperatures and wind fields in this study.


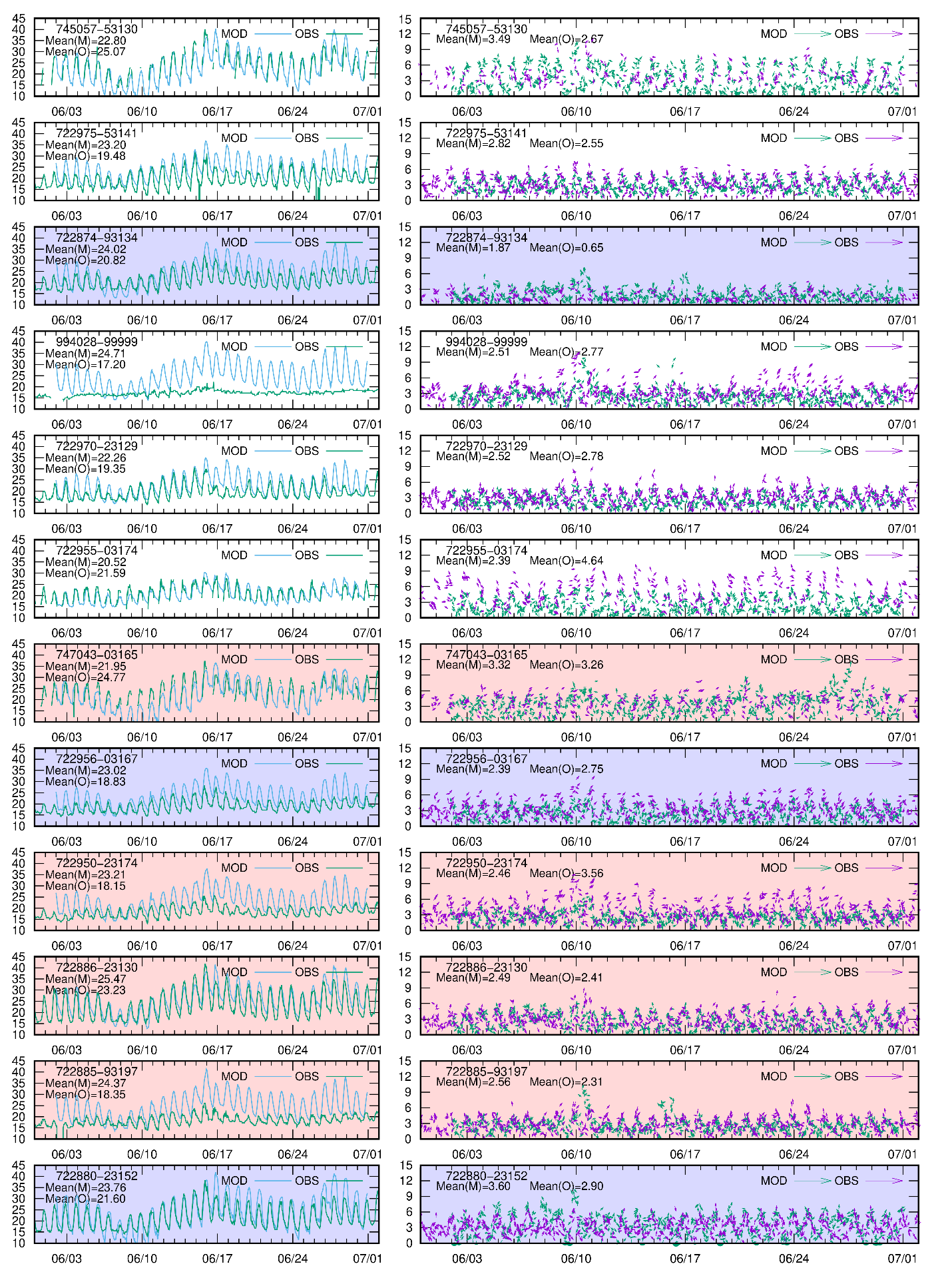


**Figure S3(H) Model performance of temperature (left) and wind (right) time series (June) in rural and urbanized areas in Los Angeles.** White indicates rural stations, blue is urban and red is sites located near airports. Station IDs are shown in each panel with the mean value of model simulations (M) and observations (O). Arrows in the right penal indicate the wind direction. Units are ºC and m/s, respectively.

Model performance was fully evaluated using hourly data for both coarse and finer domains. The model provides an overall reliable simulation when compared to observations at most monitoring sites with minor biases due to the grid resolution and the location of the monitoring stations (i.e., spatial misalignment). In addition, the lack of an updated land cover dataset in the model also induces uncertainties in calculating the heat transfer in different land cover types. This model evaluation may not be representative of the domain-wide model performance. Further efforts (e.g., a simulation with a higher horizontal resolution or using more detailed observations and land cover datasets) could be considered to improve model performance in future studies. Based on results in Figures S1, S2 and S3(A)-(G), we conclude that the model can provide statistically reliable temperature and wind simulations for heatwaves and urban heat analysis.


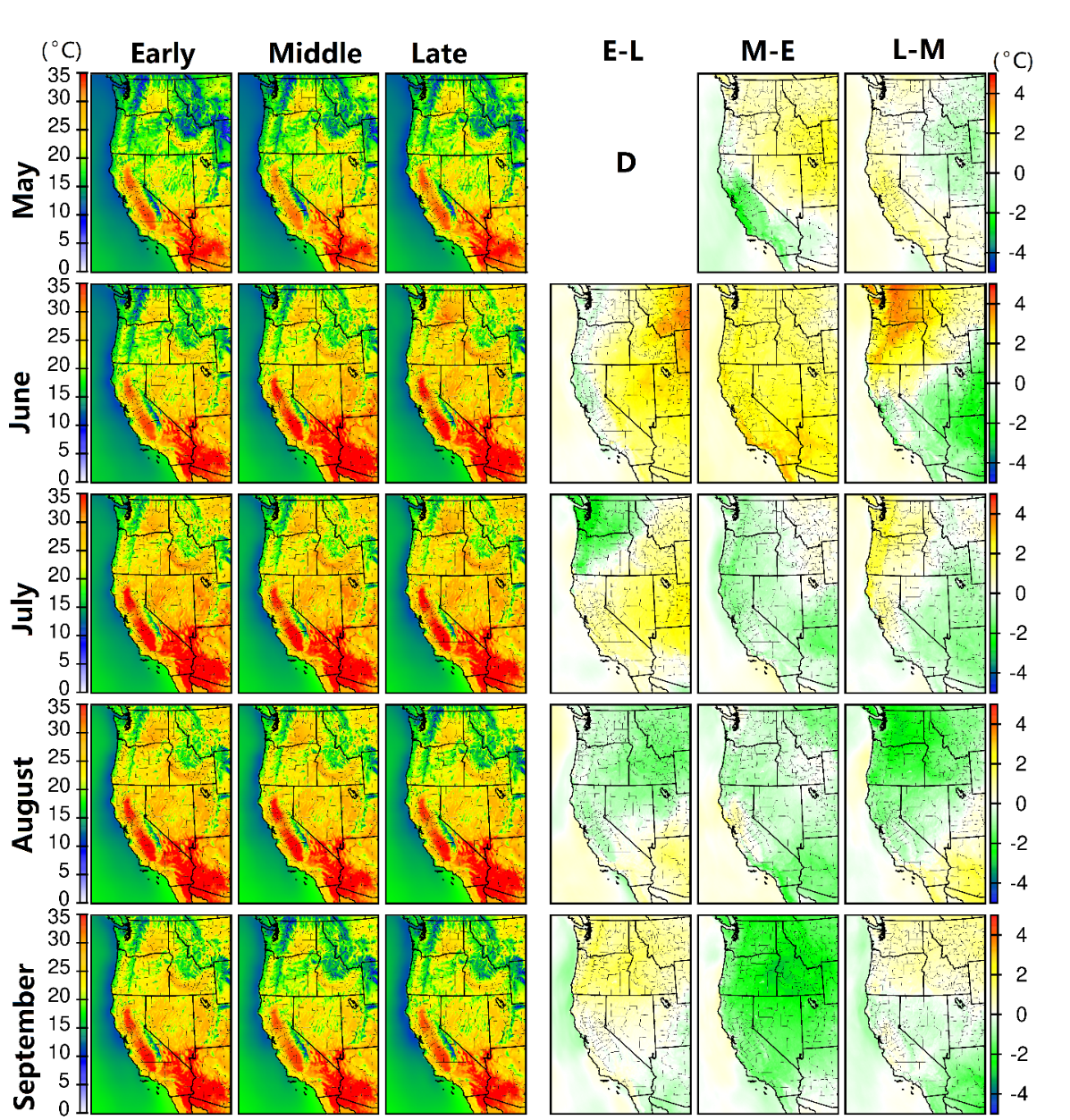


**Figure S4. Average daytime 2m-temperatures (left three columns) in the western U.S. during different episodes and their differences (right three columns).** Early (E), Middle(M) and Late(L) refer to the 1st-10th, 11th-20th and 21st to the end of each month, respectively. D refers to the differences with previous periods. Noting that the E-L means the differences between the early of the current month and the late of the previous month. Units are ºC.


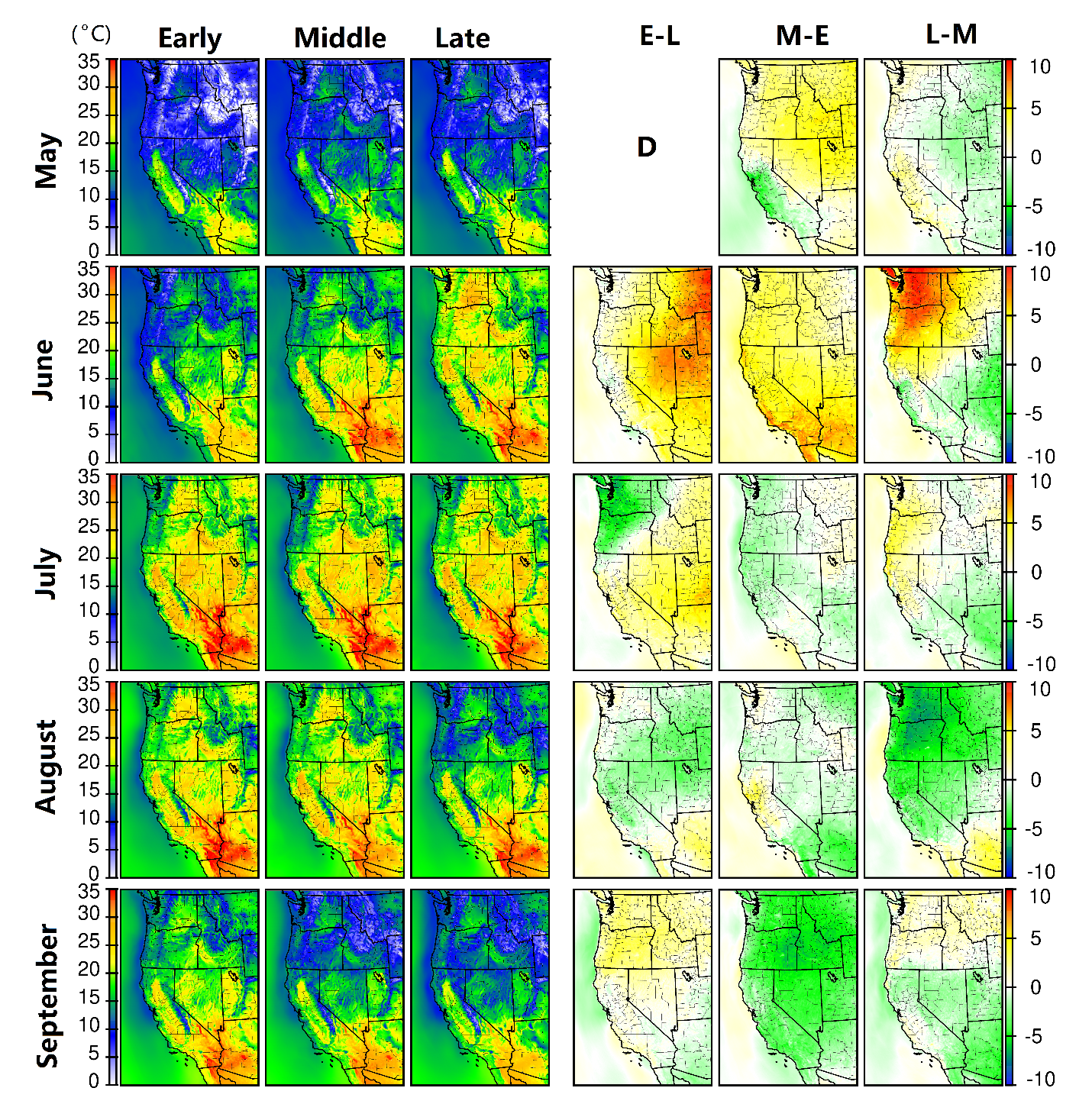


**Figure S5. Average nighttime 2m-temperatures (left three columns) in the western U.S. during different episodes and their differences (right three columns).** Early (E), Middle(M) and Late(L) refer to the 1st-10th, 11th-20th and 21st to the end of each month, respectively. D refers to the differences with previous periods. Noting that the E-L means the differences between the early of the current month and the late of the previous month. Units are ºC.


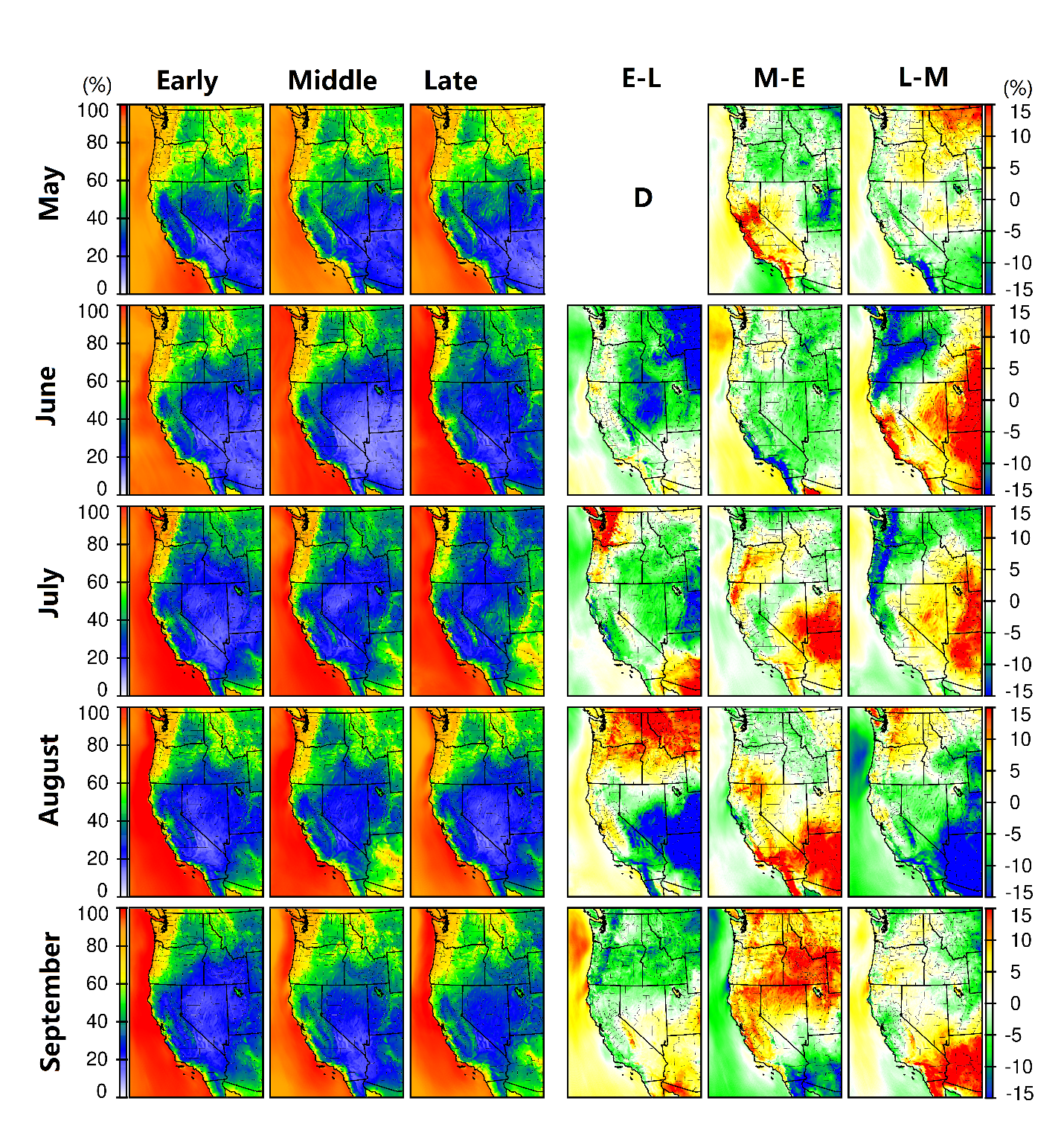


**Figure S6. Averaged 2m relative humidity in the western U.S. during different episodes and their differences.** Early (E), Middle(M) and Late(L) refer to the 1st-10th, 11th-20th and 21st to the end of each month, respectively. D refers to the differences with previous periods. Noting that the E-L means the differences between the early of the current month and the late of the previous month. Units are %.


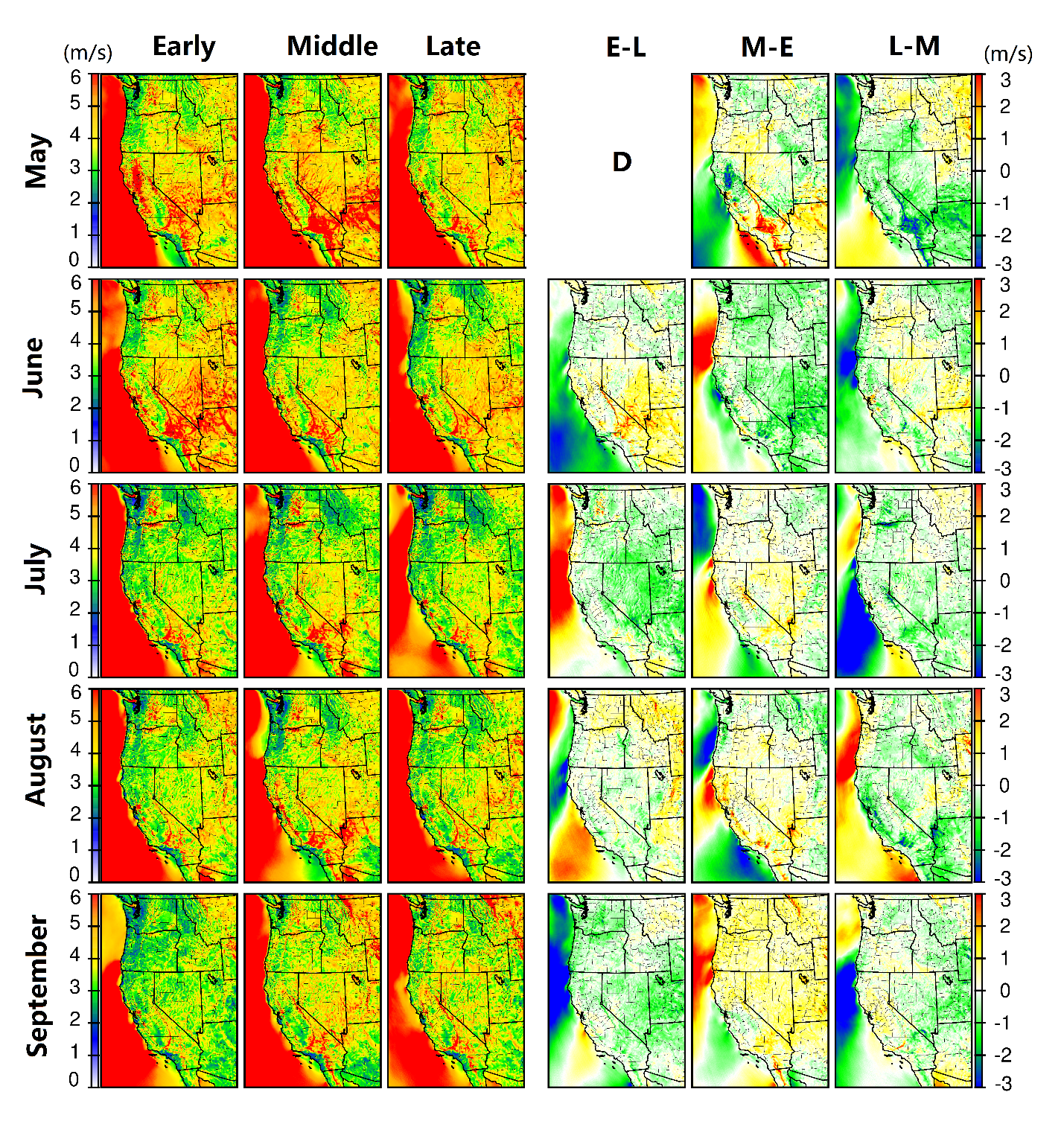


**Figure S7. Average wind speeds (left three columns) in the western U.S. during different episodes and their differences (right three columns).** Early (E), Middle(M) and Late(L) refer to the 1st-10th, 11th-20th and 21st to the end of each month, respectively. D refers to the differences with previous periods. Noting that the E-L means the differences between the early of the current month and the late of the previous month. Units are m/s.


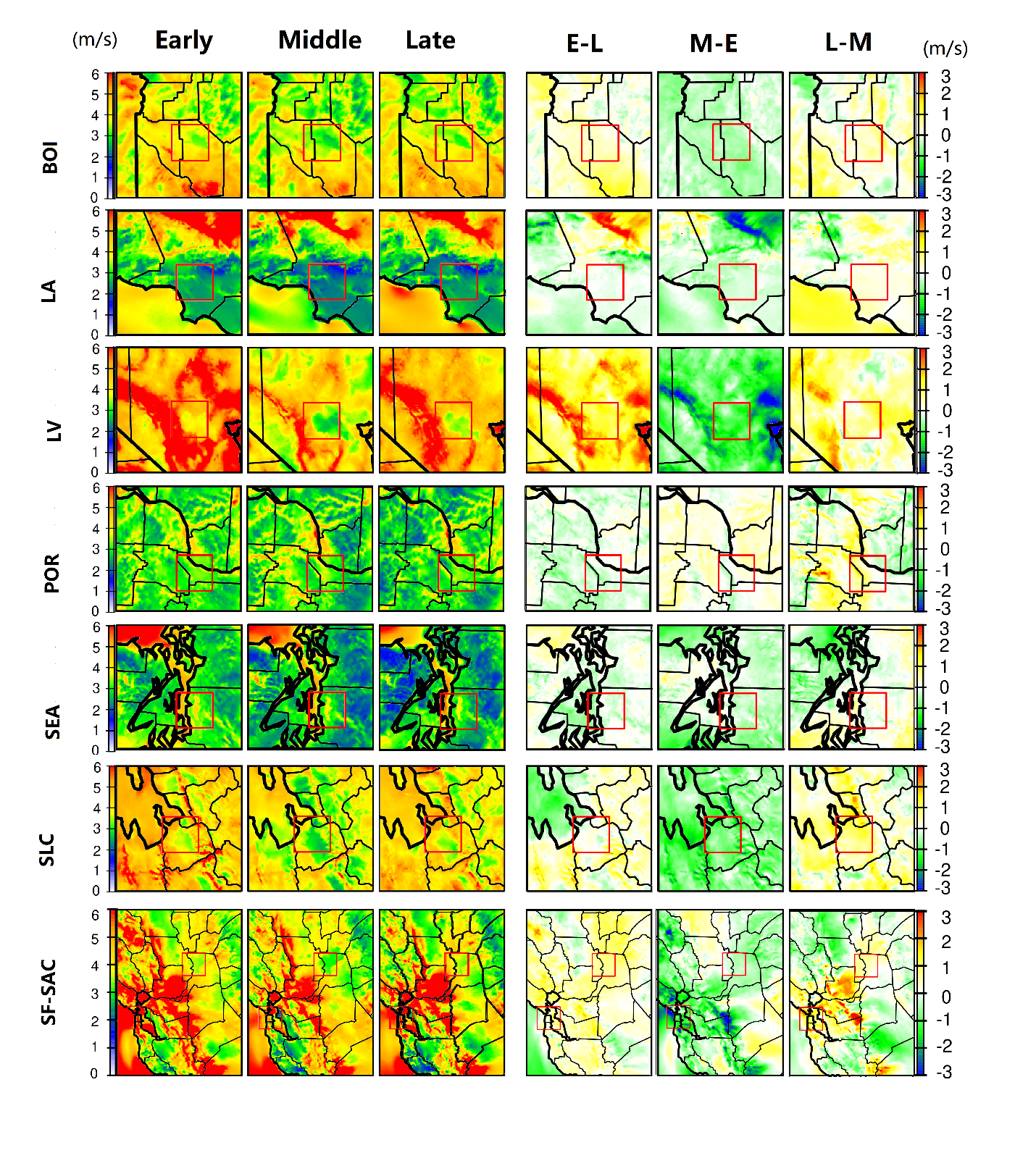


**Figure S8. Microscale wind speeds and variations over major cities.** Early (E), Middle(M) and Late(L) refer to the 1st-10th, 11th-20th and 21st to the end of June, respectively. Red squares in each panel represent the locations of major cities. SLC: Salt Lake City; LA: Los Angeles; POR: Portland; LV: Los Vegas; SEA: Seattle; BOI: Boise; SF-SAC: San Francisco and Sacramento. Units are m/s.


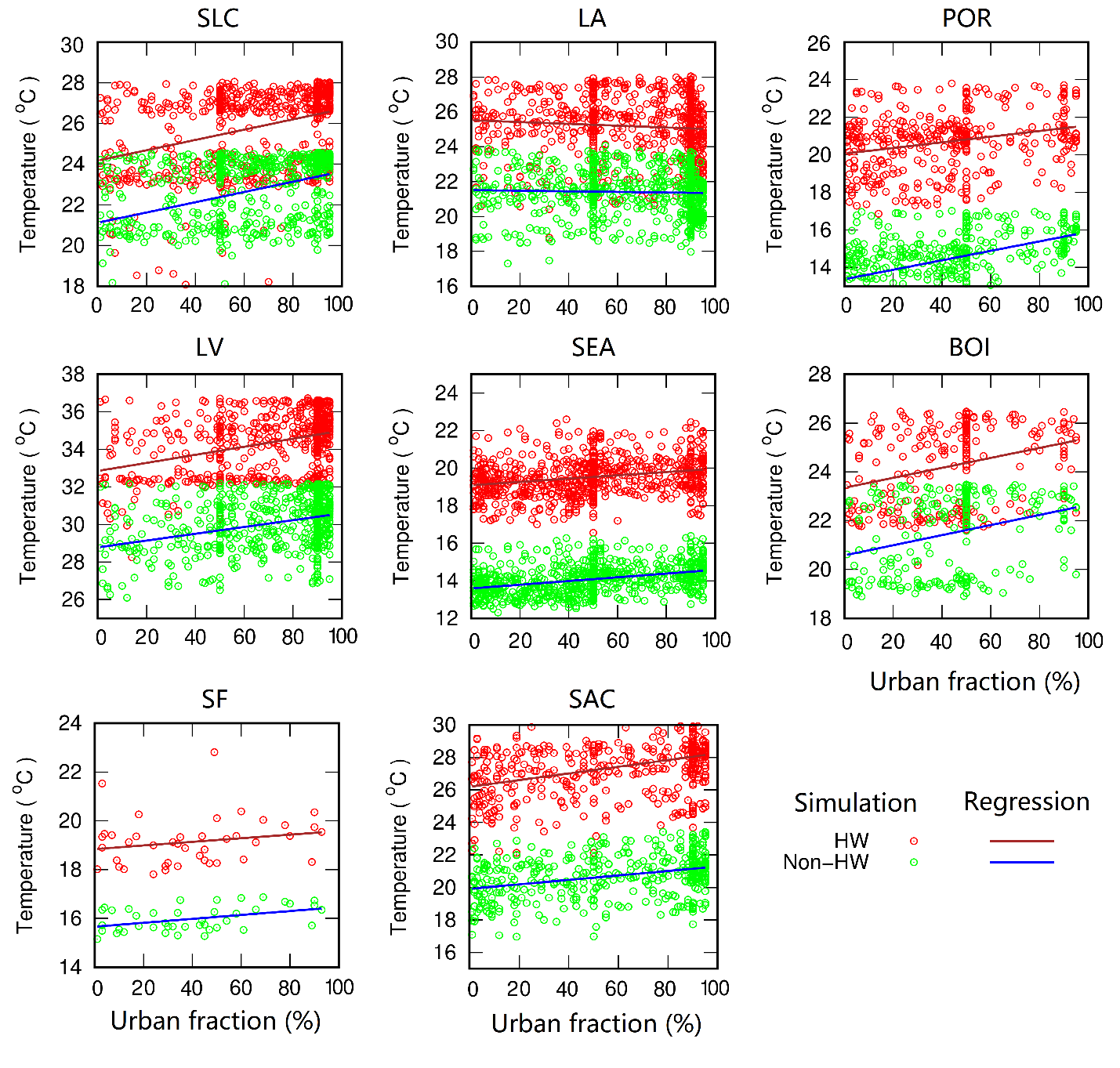
**Figure S9. Scatters plot of temperatures and urban fractions in major cities for different episodes.** Red and green circles represent the average nighttime temperatures and urban fractions in each grid in the model simulation. The brown and blue lines are linear regression fittings. The horizontal axis refers to urban fractions. Slopes, intercepts and R-values from the Pearson test can be found in the main body of the paper.


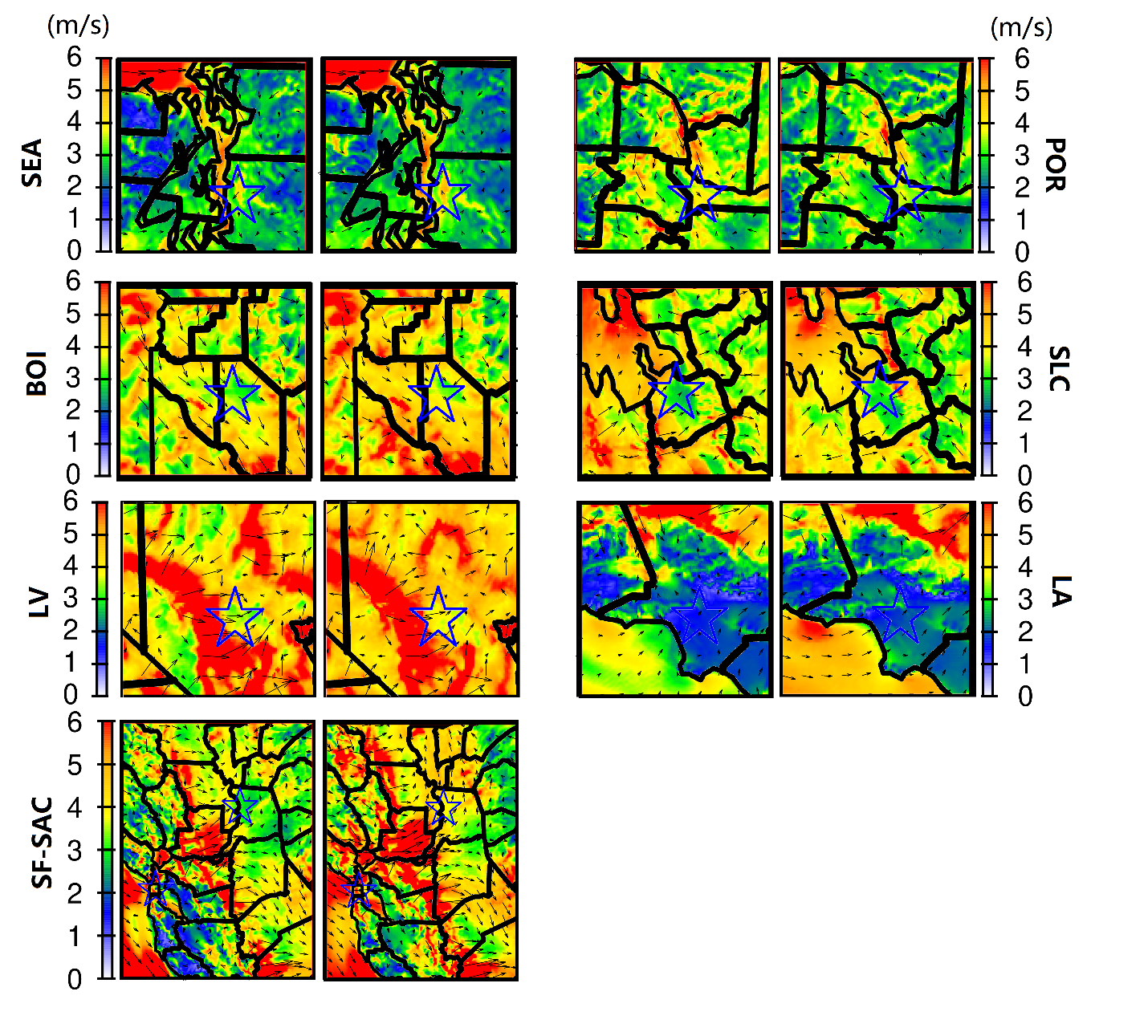


**Figure S10. Nighttime wind fields during the heatwave(left)/non-heatwave (right) periods in eight major cities in June.** *Units are m/s***.** *Note: blue stars indicate the approximate location of the cities but not the boundary of the urban regions*.

**Synoptic Scale Atmospheric Dynamic.** Table S2 shows the distances between the center of the ridge and each city. Negative values indicate being upstream of the ridge, implying that if a heatwave was present during largely negative values, likely the ridge was weak or flat, or there was another explanation for the heatwave. For example, Salt Lake City in early June 2021 was south of an incoming weak trough. However, a surface high pressure was present nearby, driving the warmer temperatures.


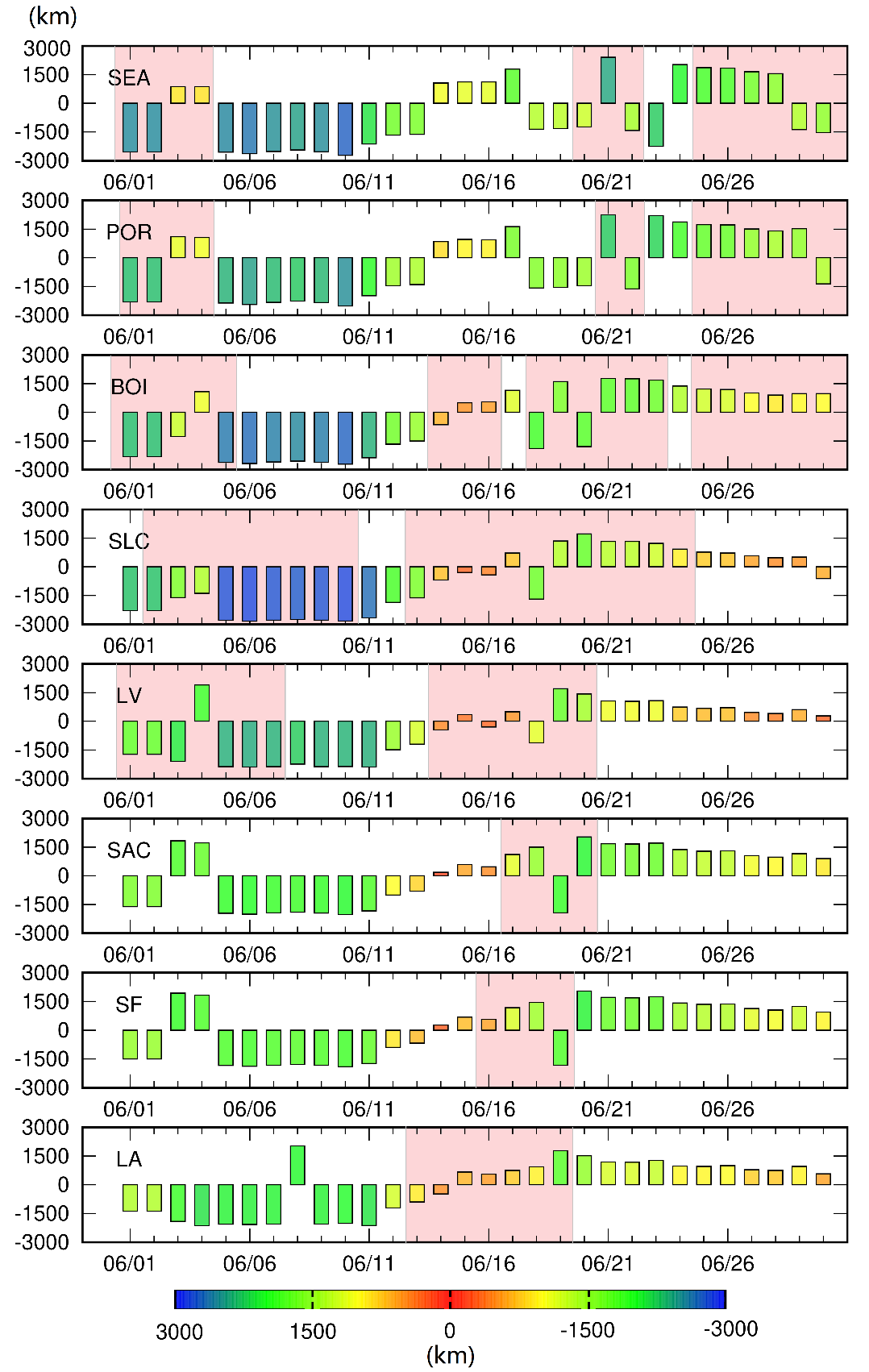


**Figure S11. The distances (km) between each city and the center of the nearest ridge in June 2021.** The light red periods indicate days when the city is experiencing a heatwave. The sign indicates if the city is upstream (negative) or downstream (positive) of the nearest ridge. Bar colors stand for the distance from close (red) to far (blue).

**Estimating UHII.** Model capabilities in capturing the urban signals are also illustrated in Figure S12. We used temperature observations obtained from MesoWest (MesoWest in the following statement) to provide higher horizontal resolution observations in calculating temperature differences between urban and rural areas. We use an average of observations from four directions to mitigate the regional differences in selecting only one urban-rural pair. The dT from the model simulations matches well with the urban/rural temperature difference from MesoWest in most cities (Seattle, Boise, Portland, Las Vegas) in magnitudes and variation trends. However, there are some exceptions. For example, during the heatwave in San Francisco, a significant drop occurred during June 17-19 were the urban/rural temperature difference is around -5 ºC. This situation occurred because the heatwave center was located over the rural area, so the urban temperatures were lower than the rural regions under the center of the heatwave. The model simulated UHII was decreasing as well during this time period but much less than the observations. This discrepancy in the UHII compared to the urban/rural temperature difference from observations is due to the regression fitting algorithm involving the suburban and low urban fraction areas, which causes less intensity in the UHI. Another significant difference occurred in Los Angeles where the city scale and built environment is large. Again, without the observations from the urban/rural transitional areas, the temperature difference will be much larger than the results from the model and UHII fitting algorithm. Additionally, these two locations are located in coastal areas and could have model biases due to sea breeze effects. Another location with a discrepancy in the urban/rural temperature differences is Salt Lake City. This is potentially due to the shrinking of the Great Salt Lake not being well captured in the model simulations. Overall, we conclude that the model and its configuration provide reliable simulations and successfully match urban signal in most cases. Model uncertainties do exist, such as differences between UHII, dT, and with temperature observations from MesoWest. Future efforts might be necessary to improve UHII estimations by using higher horizontal resolution models withupdated land cover datasets and updated urban physics.


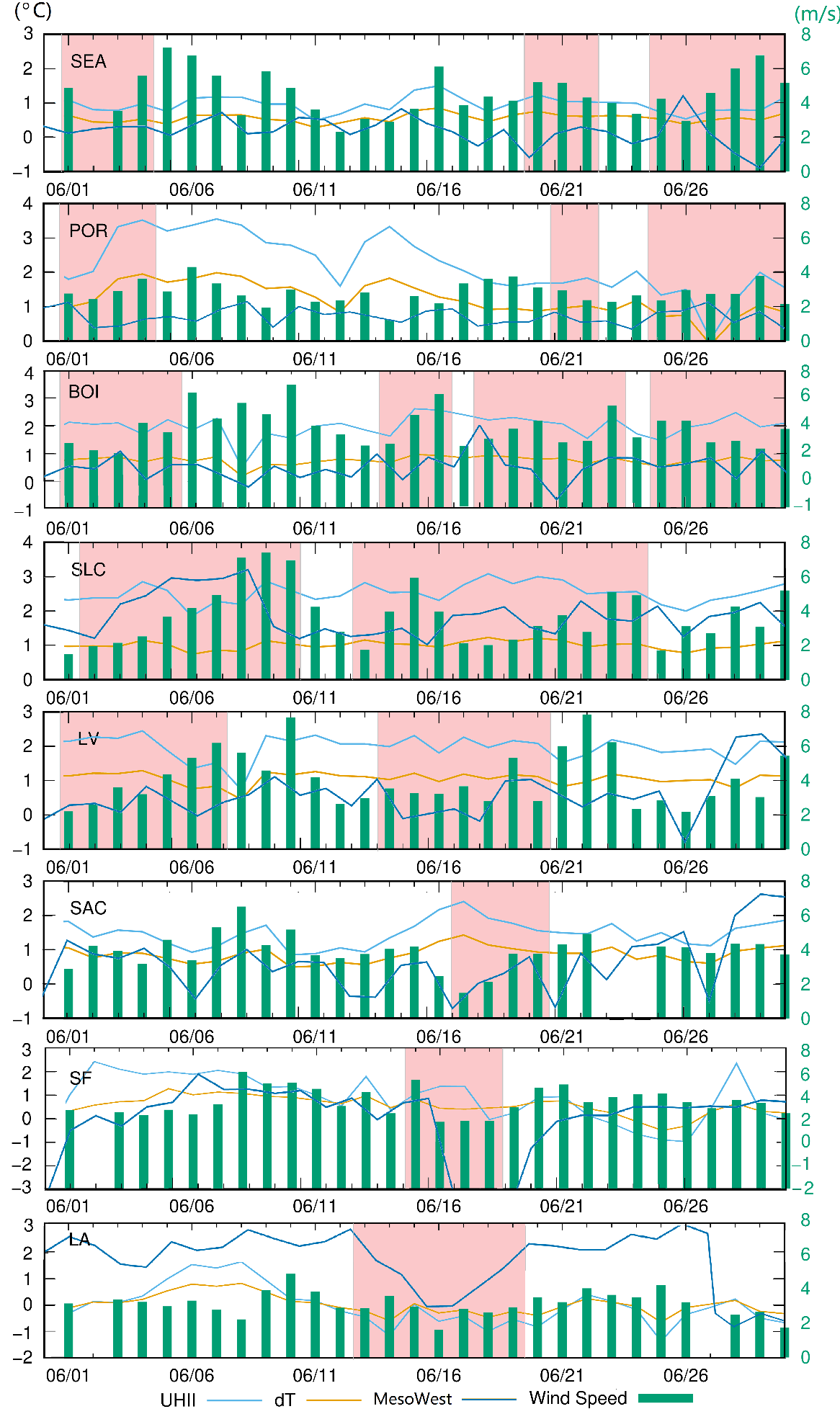


**Figure S12. Daily urban heat island intensity (UHII), mean urban vs. rural temperature difference (dT), mean nighttime temperature difference from MesoWest observations (MesoWest) and average urban nighttime wind speed in each city.** MesoWest (dark blue) and wind speed data (green) comes from observation and the UHII (light blue) and dT (orange) are obtained from model simulations. The light red shaded periods stand for heatwave episodes. Noting that some wind data is not available, which remains blank in the figure.


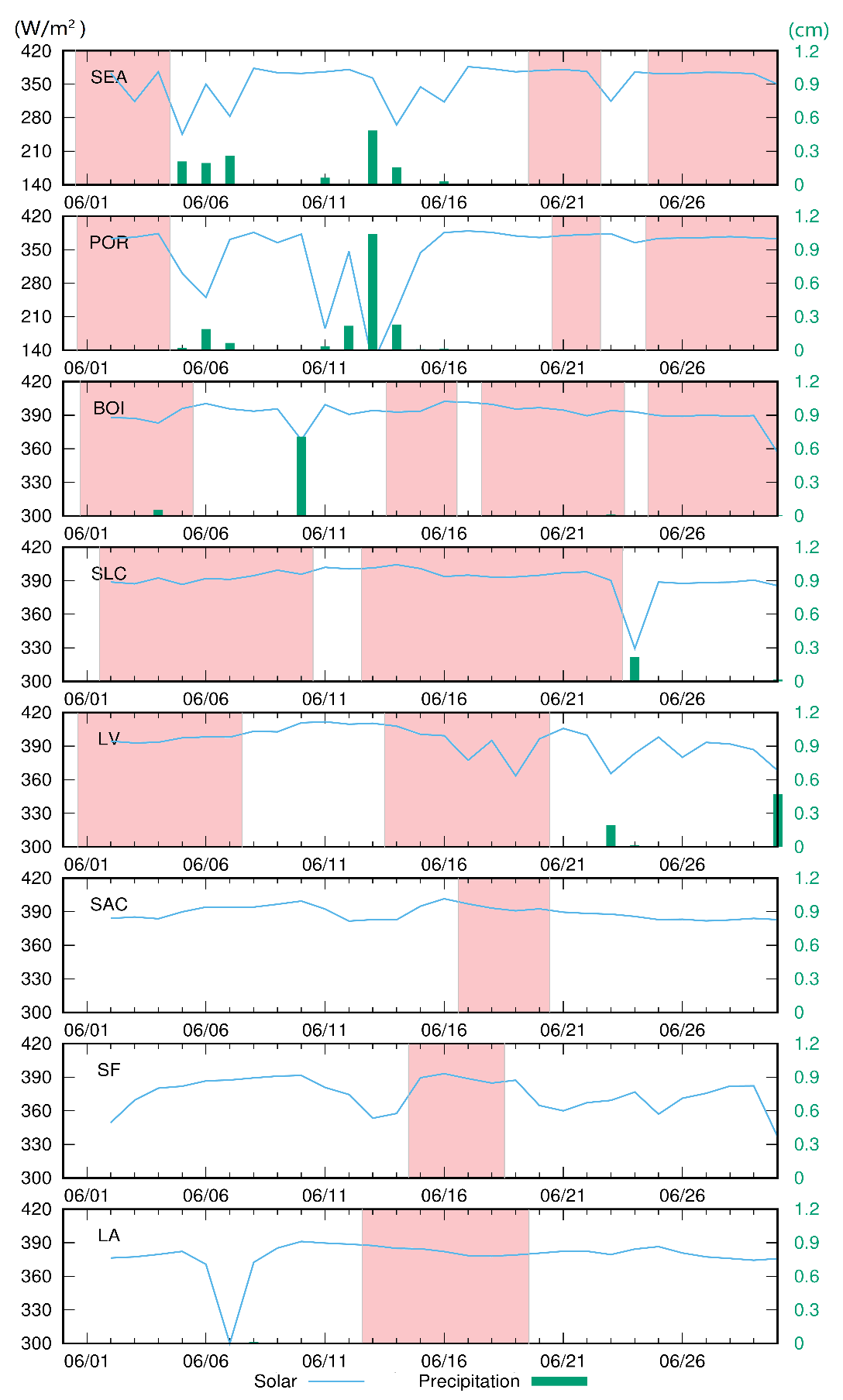


**Figure S13. Daily accumulated precipitation (cm/day) and solar radiation(shortwave) absorbed at the ground (W/m^2^) from the numerical weather prediction (WRF) model**. The light red shaded periods stand for heatwave episodes. Note: Significant reduction of the net shortwave radiation indicates cloud cover.
